# Nanofabricated Bacterial Cell Walls with Intrinsic Peroxidase‐Mimicking and Sonodynamic Activities for Cancer Combination Treatment

**DOI:** 10.1002/advs.202505310

**Published:** 2025-05-14

**Authors:** Meng Yang, Yang Meng, Junling Li, Liya Bai, Yuanyuan Cheng, Yuanyuan Liu, Mingxin Cao, Xiaoying Yang, Yinsong Wang, Yang Liu

**Affiliations:** ^1^ Key Laboratory of Immune Microenvironment and Disease (Ministry of Education) The Province and Ministry Co‐sponsored Collaborative Innovation Center for Medical Epigenetics International Joint Laboratory of Ocular Diseases (Ministry of Education) Tianjin Key Laboratory on Technologies Enabling Development of Clinical Therapeutics and Diagnostics (Theranostics) School of Pharmacy Tianjin Medical University Tianjin 300070 China; ^2^ Department of Pharmacology Tianjin Key Laboratory of Inflammatory Biology Center for Cardiovascular Diseases Key Laboratory of Immune Microenvironment and Disease (Ministry of Education) The Province and Ministry Co‐sponsored Collaborative Innovation Center for Medical Epigenetics Tianjin Medical University Tianjin 300070 China; ^3^ Department of Genetics School of Basic Medical Sciences Tianjin Medical University Tianjin 300070 China; ^4^ Department of Orthodontics Tianjin Medical University School and Hospital of Stomatology Tianjin Key Laboratory of Oral Soft and Hard Tissues Restoration and Regeneration, and Institute of Stomatology Tianjin 300070 China; ^5^ Department of Hepatobiliary Cancer Liver Cancer Center Tianjin Medical University Cancer Institute & Hospital National Clinical Research Center for Cancer Key Laboratory of Cancer Prevention and Therapy Tianjin's Clinical Research Center for Cancer Tianjin 300060 China; ^6^ Department of Hepatobiliary and Pancreatic Oncology Tianjin Cancer Hospital Airport Hospital National Clinical Research Center for Cancer Tianjin 300308 China

**Keywords:** bacterial cell walls, bacterial immunotherapy, chemotherapy, peroxidase‐mimicking activity, sonodynamic therapy

## Abstract

Bacterial cancer therapy recently has been attracting more and more attention because of its multiple functions to fight cancer. *Porphyromonas gingivalis* (Pg), a Gram‐negative pathogenic bacterium, acquires protoporphyrin IX (PpIX) and iron from heme and synthesizes abundant µ‐oxo bisheme on its cell walls (CWs). For the first time, it is found that the CWs extracted from Pg has intrinsic peroxidase (POD)‐mimicking and sonodynamic activities owing to the presence of µ‐oxo bisheme. In this study, the CWs of Pg are nanofabricated to form the CW vesicles (CWV) containing a large amount of lipopolysaccharide (LPS) and further encapsulated doxorubicin (DOX) to prepare DOX‐loaded CWV (DOX@CWV), hoping to eradicate cancer by combining sonodynamic therapy (SDT), chemotherapy, and bacterial immunotherapy. The results confirmed that DOX@CWV can catalyze the conversion of H_2_O_2_ into O_2_ and consume the reduced glutathione (GSH), and thus greatly boost their own sonodynamic performance upon ultrasonic irradiation. Both in vitro and in vivo, DOX@CWV efficiently inhibited cancer growth by combining SDT and chemotherapy, and also exerted synergistic anticancer immune effects of bacterial immunotherapy and SDT. In summary, the findings not only contribute a promising bacterial therapeutic agent but also provide a combination strategy for clinical cancer treatment.

## Graphical abstract

Bacterial cell walls (CWs) extracted from *Porphyromonas gingivalis* are nanofabricated to form the CW vesicles (CWV), which have intrinsic peroxidase‐mimicking and sonodynamic activities due to the presence of µ‐oxo bisheme and meanwhile retain a large amount of lipopolysaccharide. Doxorubicin is efficiently encapsulated into CWV and thus can combine sonodynamic therapy, chemotherapy, and bacterial immunotherapy to suppress cancer growth and metastasis.

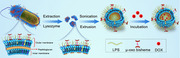

## 1 Introduction

Bacterial cancer therapy, which utilizes the living, attenuated or killed bacteria, and bacterial components as therapeutic agents, has long been applied to fight cancer directly or facilitate other cancer treatments.^[^ ^1^, ^2^ ^]^ Owing to the presence of large amounts of pathogen‐associated molecular patterns (PAMPs), bacterial therapeutic agents can activate a broad range of immune cells that express pattern recognition receptors (PRRs), thus initiate strong immune responses to overcome tumor immune escape.^[^ ^3^, ^4^ ^]^ Lipopolysaccharide (LPS), derived from the cell outer walls of Gram‐negative bacteria, has been proved to be effective in the improvement of malignant tumors' clinical therapy through recovering the immune competence of small intestines and boosting the activity of macrophages.^[^ ^5^ ^]^ Besides the immunoadjuvant activity, some natural bacteria or genetically engineered bacteria can also secret bioactive substances such as toxins and enzymes to exert synergistic anticancer effects.^[^ ^6^, ^7^ ^]^ Recently, we developed a bacterial nanomedicine from *Porphyromonas gingivalis* (Pg), in which cells contain photosensitizer protoporphyrin IX (PpIX), thus combined photodynamic therapy (PDT) and bacterial immunotherapy to inhibit cancer growth and metastasis.^[^ ^8^ ^]^

Pg is a Gram‐negative pathogenic bacterium in oral cavity and acquires PpIX and iron from heme for survival and virulence.^[^ ^9^ ^]^ On its cell walls (CWs), Pg synthesizes a black pigment composed of µ‐oxo bisheme ([Fe(III)PpIX]_2_O) by using PpIX and iron to protect itself from the host neutrophil attack.^[^ ^10^ ^]^ For the first time, we found that the CWs extracted from Pg had significant peroxidase (POD)‐mimicking and sonodynamic activities owing to the presence of [Fe(III)PpIX]_2_O. Considering that hypoxia and H_2_O_2_ accumulation play key roles in tumorigenesis, development, and metastasis,^[^ ^11^, ^12^ ^]^ the POD‐mimicking activity of the Pg's CWs will theoretically contribute to cancer treatments by catalyzing the decomposition of H_2_O_2_ into O_2_. This applies in particular to the treatments needing the involvement of O_2_, such as PDT and sonodynamic therapy (SDT).^[^ ^13^, ^14^ ^]^ In recent years, SDT has gained more attention than PDT in the field of cancer research due to the strong tissue‐penetration ability of ultrasound.^[^ ^15^ ^]^ Besides O_2_ and ultrasound, sonosensitizers are another key element for efficient SDT.^[^ ^16^ ^]^ Upon ultrasonic (US) irradiation, sonosensitizers can induce the generation of cytotoxic reactive oxygen species (ROS) to destroy cancer cells and remodel the tumor environment (TME).^[^ ^17^, ^18^ ^]^ Some studies have shown that metalloporphyrin complexes are excellent sonosensitizers for SDT.^[^ ^19^, ^20^ ^]^ In view of the structure character of [Fe(III)PpIX]_2_O, we believe that the Pg's CWs can be used as a biomimetic sonosensitizer for cancer SDT. As a POD‐mimicking catalyst, [Fe(III)PpIX]_2_O can also oxidize the reduced glutathione (GSH), one of the most important scavengers of ROS in cancer cells.^[^ ^21^ ^]^ This will help maintain the high levels of intracellular ROS and thus boost the anticancer efficacy of SDT.^[^ ^22^ ^]^ In this study, CW vesicles (CWV) integrate sonosensitizing and peroxidase‐mimicking functions, enhancing therapeutic efficacy and offering a versatile cancer treatment platform. Unlike existing sonosensitizers, which suffer from hydrophobicity, phototoxicity, and low delivery efficiency,^[^ ^23^, ^24^ ^]^ and peroxidase mimics, which face stability and specificity challenges,^[^ ^25^ ^]^ Pg's CWs provide a unique advantage. With sonodynamic activity, POD‐mimicking properties, and drug delivery capability, Pg's CWs hold strong potential as an effective sonosensitizer for cancer SDT.

**Scheme 1:**
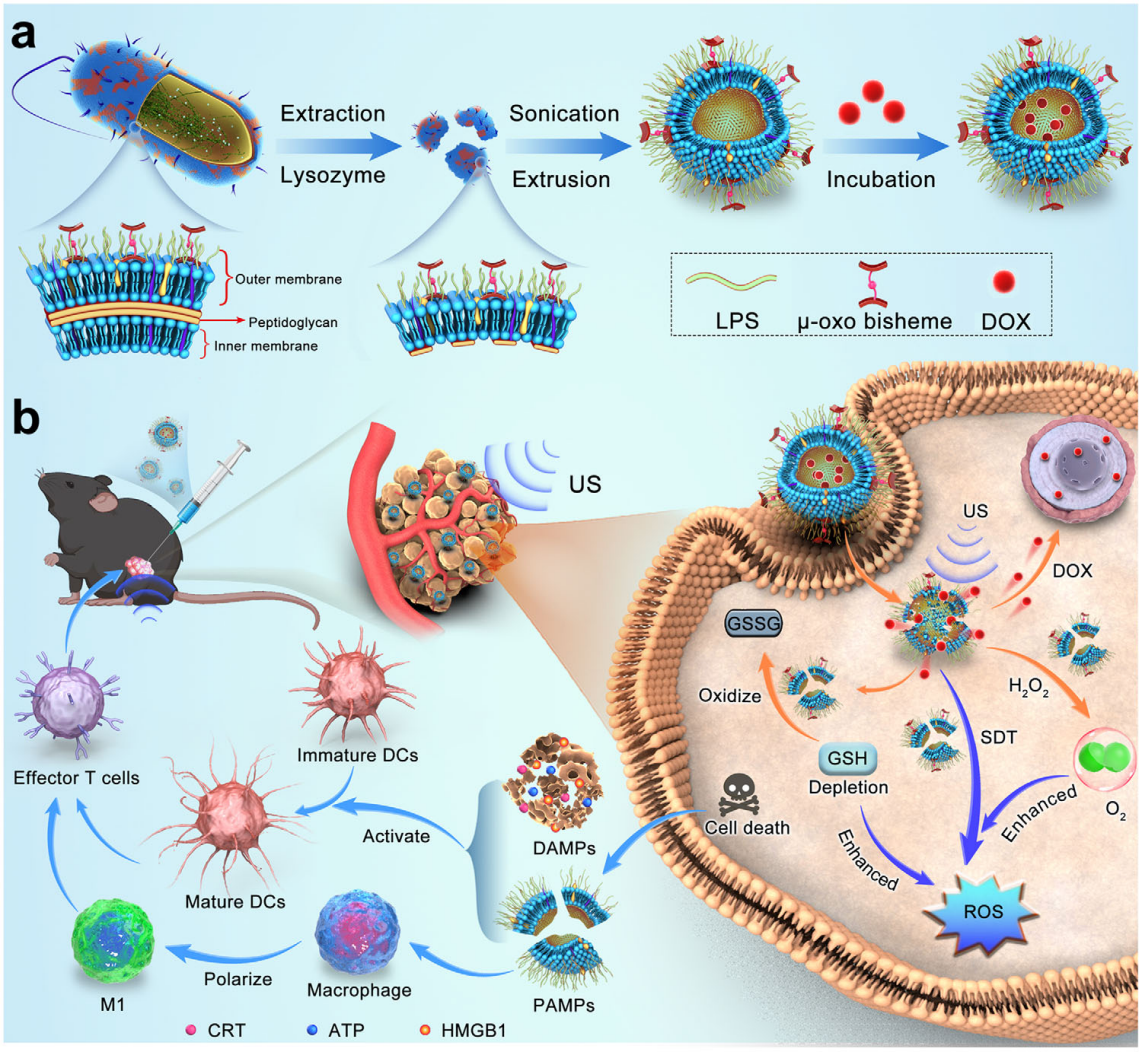
Schematic illustrations for a) preparation of DOX@CWV and b) in vivo synergistic anticancer effects of DOX@CWV‐mediated combination treatments of SDT, chemotherapy, and bacterial immunotherapy.

In this study, we obtained CWs containing the bacterial outer membrane by lysozyme digestion in the peptidoglycan layer of the bacterial cell wall. The CWs with intrinsic POD‐mimicking and sonodynamic activities are further nanofabricated to form the CWV through sonication and extrusion. Doxorubicin (DOX), a clinical broad‐spectrum chemotherapeutic agent, is next encapsulated into CWV by simple incubation to obtain DOX‐loaded CWV (DOX@CWV) for cancer combination treatment (**Scheme** **1**a). In view of the wide applications of ultrasonic modality in stomatology,^[^ ^26^ ^]^ oral cancer is chosen as a model of solid tumors to investigate the synergistic anticancer effects of DOX@CWV‐mediated combination treatment of SDT, chemotherapy, and bacterial immunotherapy. Scheme 1b illustrates the mechanisms of synergistic anticancer effects in vivo. Upon US irradiation, DOX@CWV exert sonodynamic performance to trigger the generation of cytotoxic ROS, thereby inducing the immunogenic cell death (ICD) of cancer cells. In the meantime, the POD‐mimicking activity of DOX@CWV promotes the in‐situ production of O_2_ by catalytically decomposing H_2_O_2_ in the TME and also consume the intracellular GSH to facilitate SDT. Upon US irradiation, DOX is acceleratedly released from DOX@CWV and exerts cytotoxic activity to induce the death of cancer cells, thereby further enhancing the anticancer efficacy of SDT through combining chemotherapy. Damage associated molecular patterns (DAMPs) released from the dying cancer cells and LPS existing in Pg's CWs cooperatively initiate potent anticancer immune responses, including the activation and maturation of dendritic cells (DCs), the differentiation of effector T cells, and the polarization of M1 macrophages, thus to suppress cancer growth and metastasis. In this study, we will systematically investigate the synergistic anticancer effects of DOX@CWV‐mediated combination treatment against oral cancer both in vitro and in vivo.

## 2 Results and Discussion

### 2.1 Preparation, Characterization, and In Vitro Drug Release of DOX@CWV

**Figure 1:**
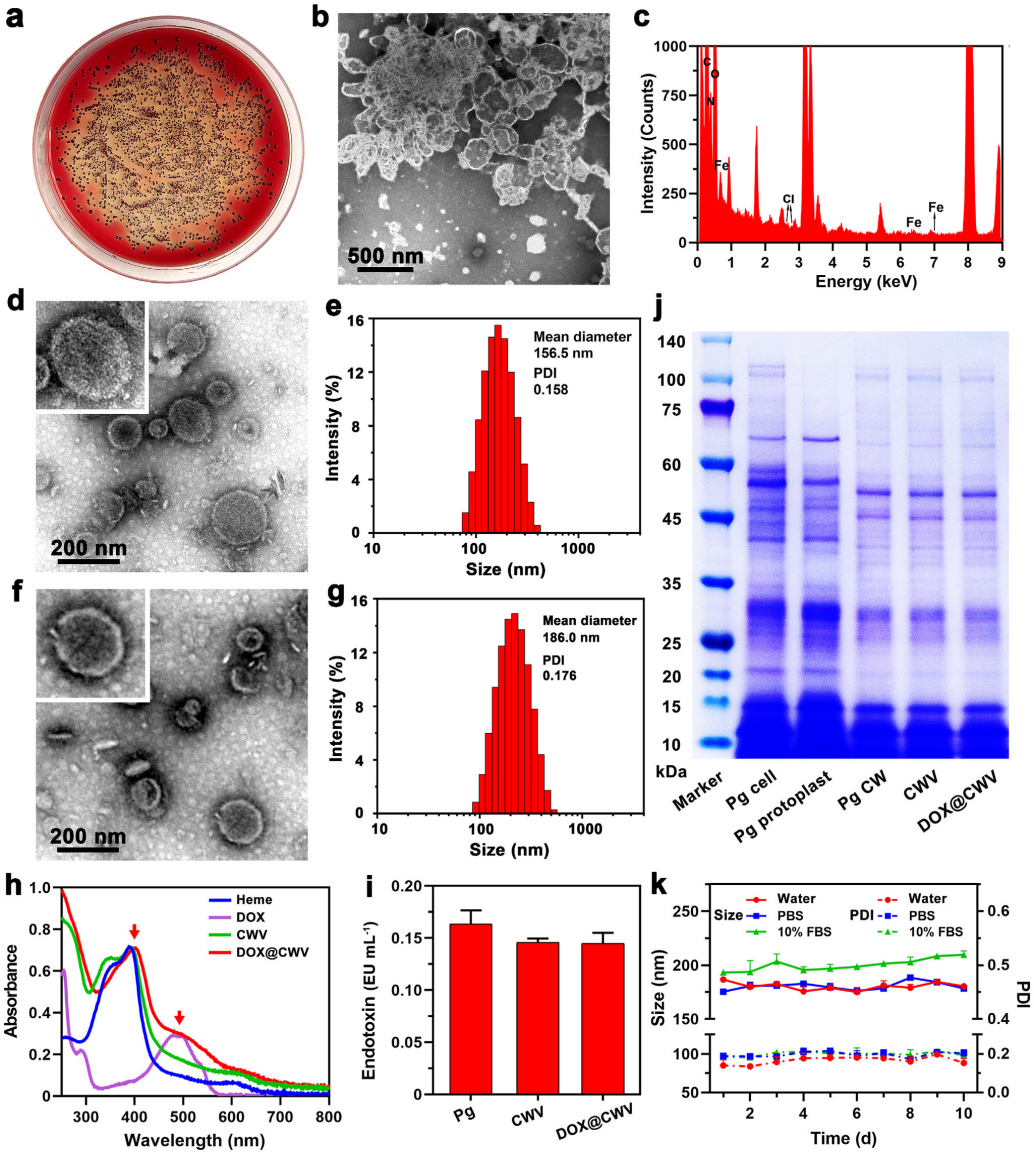
Preparation and characterization of DOX@CWV. a) Photograph of Pg colonies grown on sheep blood agar plate. b) TEM image and c) EDS energy spectrum of bacterial CWs extracted from Pg. Scale bar: 500 nm. d) TEM image and e) size distribution of CWV. Scale bar: 200 nm. f) TEM image and g) size distribution of DOX@CWV. Scale bar: 200 nm. h) UV‐vis spectra of heme, CWV, and DOX@CWV at the same concentrations of CWV and DOX. i) Endotoxin contents of Pg, CWV, and DOX@CWV. Data are presented as mean values ± SD (*n* = 3). j) SDS–PAGE profile of Coomassie brilliant blue‐stained proteins extracted from Pg cells, protoplasts, CWs, CWV, and DOX@CWV. k) Sizes and PDIs of DOX@CWV within 10 d of storage in deionized water, PBS, and 10% FBS. Data are shown as mean values ± SD (*n* = 3).

Bacterial CWs were extracted from 1 × 10^4^ colony‐forming units (CFUs) of Pg cultured on sheep blood plates (**Figure** **1**a) using an improved lysozyme method according to the previous report.^[^ ^27^ ^]^ Lysozyme hydrolyzed peptidoglycan in Pg's CWs by breaking the 1,4‐beta‐linkages between N‐acetylmuramic acid and N‐acetylglucosamine, separating the CWs with bacterial outer membranes from the cell plasma membranes. Bacterial CWs were obtained after centrifugation to remove protoplasts and further purified by ultrafiltration. Under a transmission electron microscopy (TEM), the membrane fragments of bacterial CWs were observed clearly (Figure 1b). Energy‐dispersive X‐ray spectroscopy (EDS) elemental mapping demonstrated the presence of Fe in bacterial CWs (Figure 1c). Inductively coupled plasma‐mass spectrometry (ICP‐MS) was further used to detect the content of Fe in bacterial CWs and the result was 0.30 ± 0.02 mg g^−1^. After ultrasonic disruption and extrusion through a 200‐nm pore size filter, bacterial CWs formed spherical CWV (Figure 1d). In aqueous medium, CWV exhibited a hydraulic diameter of 156.5 nm with a polydispersity index (PDI) of 0.158 (Figure 1e), indicating their good dispersibility.

DOX, a clinical broad‐spectrum chemotherapeutic agent that can penetrate the cell membranes,^[^ ^28^ ^]^ was next encapsulated into CWV after incubation under stirring for 1 h to prepare DOX@CWV. By varying the input amount of DOX, DOX@CWV with different DOX encapsulation efficiencies were obtained (Table S1, Supporting Information), indicating that DOX@CWV can be prepared on‐demand. In the following experiments, 30 µg of DOX was encapsulated into CWV sourced from 1.5 × 10^7^ Pg cells to prepare DOX@CWV, in which the encapsulation efficiency of DOX was 85.4 ± 2.1%. Compared with CWV, DOX@CWV maintained spherical shape (Figure 1f), but their hydraulic diameter and PDI were increased to 186.0 nm and 0.176, respectively (Figure 1g). The ultraviolet‐visible (UV‐vis) spectra of various samples are shown in Figure 1h. CWV displayed typical absorption peaks consistent with heme, suggesting that they can be quantitatively analyzed as compared to heme. Furthermore, DOX@CWV displayed the characteristic absorption peaks of both CWV and DOX. The contents of LPS were detected using the bacterial endotoxin detection kit. The LPS contents of CWV and DOX@CWV were only slightly reduced while compared to Pg (Figure 1i), meaning that LPS was efficiently retained during the preparation process. We further used SDS‐polyacrylamide gel electrophoresis (SDS‐PAGE) and Coomassie blue staining to evaluate the protein components of DOX@CWV. As shown in Figure 1j, CWV and DOX@CWV exhibited almost identical protein bands to the Pg's CWs. These results confirmed that DOX@CWV were successfully prepared using the method in this study.

We further evaluated the colloidal stabilities of DOX@CWV in deionized water, phosphate buffer saline (PBS), and 10% fetal bovine serum (FBS) by monitoring the changes of their particle sizes and PDIs within 10 d of storage at 4 °C. As shown in Figure 1k, DOX@CWV exhibited relatively constant particle sizes and PDIs in these aqueous media, suggesting their excellent colloidal stabilities in the subsequent use process. Given that the efficient release is important for DOX to exert cytotoxic effect, we further measured the release ability of DOX from DOX@CWV under different conditions. Whether preprocessing with or without 5‐minute US irradiation (1 MHz, 1.5 W cm^−2^), DOX showed a significant pH‐responsive release behavior from DOX@CWV. While the pH was decreased from 7.4 to 5.5, the cumulative release ratios of DOX were increased from 20.4% to 45.5% at 24 h (Figure S1a, Supporting Information), and increased from 30.3% to 58.6% after US irradiation (Figure S1b, Supporting Information). It indicated that US irradiation also markedly promoted the release of DOX. In view of the acidic condition of the TME and the focusability of US irradiation, the controlled release of DOX from DOX@CWV at the tumor site is fully realizable, thus will be conducive to improving the anticancer efficacy and reduce the side toxicity of DOX.

### 2.2 POD‐Mimicking and Sonodynamic Activities of DOX@CWV in Aqueous Solution

**Figure 2:**
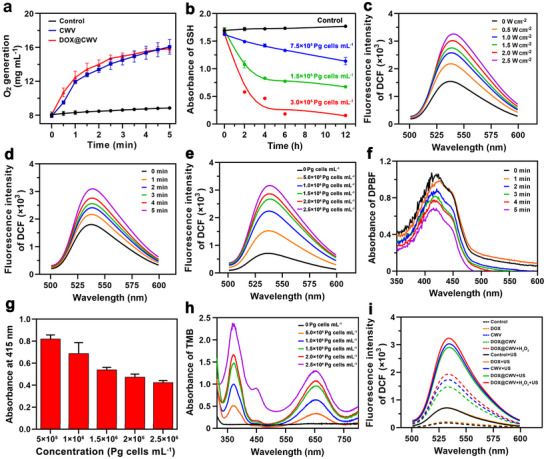
POD‐mimicking and sonodynamic activities of DOX@CWV in aqueous solution. a) O_2_ production levels in the solutions of CWV and DOX@CWV after adding 100 µmol L^−1^ H_2_O_2_ (+H_2_O_2_). The concentration of CWV was ≈1.5 × 10^6^ Pg cells mL^−1^. Data are presented as mean values ± SD (*n* = 3). b) GSH consumption levels in DOX@CWV solutions at different concentrations of CWV. Fluorescence spectra of DCF produced in DOX@CWV solutions upon US irradiation at c) different power intensities, d) different US time, and e) different concentrations of CWV. f) UV‐vis absorption spectra of DPBF in DOX@CWV solutions after different times of US irradiation. g) DPBF absorbances at 415 nm in DOX@CWV solutions with different concentrations of CWV after 5‐minute US irradiation. Data are presented as mean values ± SD (*n* = 3). h) UV‐vis absorption spectra of TMB in DOX@CWV solutions after 5‐minute US irradiation at different concentrations of CWV. i) Fluorescence spectra of DCF produced in the solutions of DOX, CWV, DOX@CWV, and DOX@CWV+H_2_O_2_ (‒ US), and their combination with 5‐minute US irradiation (+ US). US irradiation (1 MHz, 1.5 W cm^−2^) was performed for 5 min.

Owing to the presence of [Fe(III)PpIX]_2_O, DOX@CWV theoretically has the POD‐mimicking and sonodynamic activities. Here, we evaluated the POD‐mimicking activity of DOX@CWV by detecting the production of O_2_ from H_2_O_2_ and the consumption of GSH in PBS. Compared to the control, CWV and DOX@CWV (the concentration of CWV was approximately 5 × 10^6^ Pg cells mL^−1^) significantly promoted the production of O_2_ in PBS supplemented with 100 µmol L^−1^ of H_2_O_2_ (**Figure** **2**a), demonstrating that these POD‐mimicking vesicles catalytically decomposed H_2_O_2_ into O_2_. Ellman's reagent, a color‐developing reagent for quantitative analysis of thiols,^[^ ^29^ ^]^ was used to detect the consumption of GSH. In PBS containing 0.25 mmol L^−1^ of GSH, the concentrations of thiols rapidly decreased while incubation with DOX@CWV, and moreover, the decrease was markedly accelerated when the concentration of CWV was increased from 7.5 × 10^5^ to 3 × 10^6^ Pg cells mL^−1^ (Figure 2b). DOX@CWV clearly demonstrated POD‐mimicking activity, effectively consuming GSH.

Next, we evaluated the sonodynamic activity of DOX@CWV upon US irradiation. 2′,7′‐Dichlorodihydrofluorescein (DCFH) that is hydrolyzed from 2′,7′‐dichlorofluorescein diacetate (DCFH‐DA) can react with ROS in aqueous solution to form the highly fluorescent 2′,7′‐dichlorofluorescein (DCF).^[^ ^30^ ^]^ Thereby, DCFH was used as a fluorescence probe to detect the generation of ROS triggered by DOX@CWV in PBS after US irradiation. When the concentration of CWV was ≈1.5 × 10^6^ Pg cells mL^−1^, DOX@CWV accelerated the generation levels of ROS in US irradiation power and time dependent manners (Figure 2c,d). Given that US irradiation with a frequency of 1 MHz may cause cytotoxicity at a power density above 2 W cm^−2^ (Figure S2, Supporting Information), 1.5 W cm^−2^ was chosen to perform US irradiation in the following experiments. Both the aqueous solution and cellular‐level experiments demonstrated a strong effect with 5 min of ultrasound irradiation. Therefore, subsequent experimental conditions were conducted using an intensity of 1.5 W cm^−2^ for 5 min. After 5 min of US irradiation, DOX@CWV triggered the generation of ROS in a CW concentration‐dependent manner (Figure 2e), confirming their sonodynamic activity. We further explored the types of generated ROS by using the specific molecular probes. 1,3‐Diphenylisobenzofuran (DPBF) was used as an indicator for exploring the single‐linear state oxygen (^1^O_2_).^[^ ^31^ ^]^ DPBF displayed increasingly reduced absorption curve within 5 min of US irradiation (Figure 2f) and decreased absorbance at 415 nm with the increase of CW concentration (Figure 2g), demonstrating the generation of ^1^O_2_ triggered by DOX@CWV. 3,3′,5,5′‐Ttetramethylbenzidine (TMB) was utilized to detect the generation of hydroxyl radicals (⋅OH).^[^ ^32^ ^]^ As shown in Figure 2h, DOX@CWV gradually enhanced the absorbance of TMB at 652 nm with the increase of CW concentration, demonstrating that DOX@CWV exerted the sonodynamic activity to induce the generation of ⋅OH. From these results, it can be deduced that ⋅OH and ^1^O_2_ are two main types of ROS generated from the sonodynamic activity of DOX@CWV.

Finally, DCFH was applied to compare the generation levels of ROS in various sample solutions upon 5‐minute US irradiation (1 MHz, 1.5 W cm^−2^), and the results are shown in Figure 2i. CWV and DOX@CWV both greatly promoted the generation of ROS in PBS after US irradiation, whereas the produced ROS in PBS (the control) and DOX solution were only enhanced slightly. These results confirmed that CWV and DOX@CWV had significant sonodynamic activity and can be applied as the sonosensitizers for cancer SDT. In the meantime, we evaluated the sonodynamic activity of DOX@CWV in PBS containing 100 µmol L^−1^ of H_2_O_2_. With or without US irradiation, H_2_O_2_ obviously enhanced the generations of ROS induced by DOX@CWV to varying degrees. This confirmed that DOX@CWV exhibited POD‐mimicking activity, catalytically decomposing H_2_O_2_ into O_2_, thereby amplifying their sonodynamic activity. Given the relatively high levels of H_2_O_2_ in the TME, DOX@CWV great potential as an effective sonosensitizer for cancer SDT.

### 2.3 POD‐Mimicking and Sonodynamic Activities of DOX@CWV in Cancer Cells

**Figure 3:**
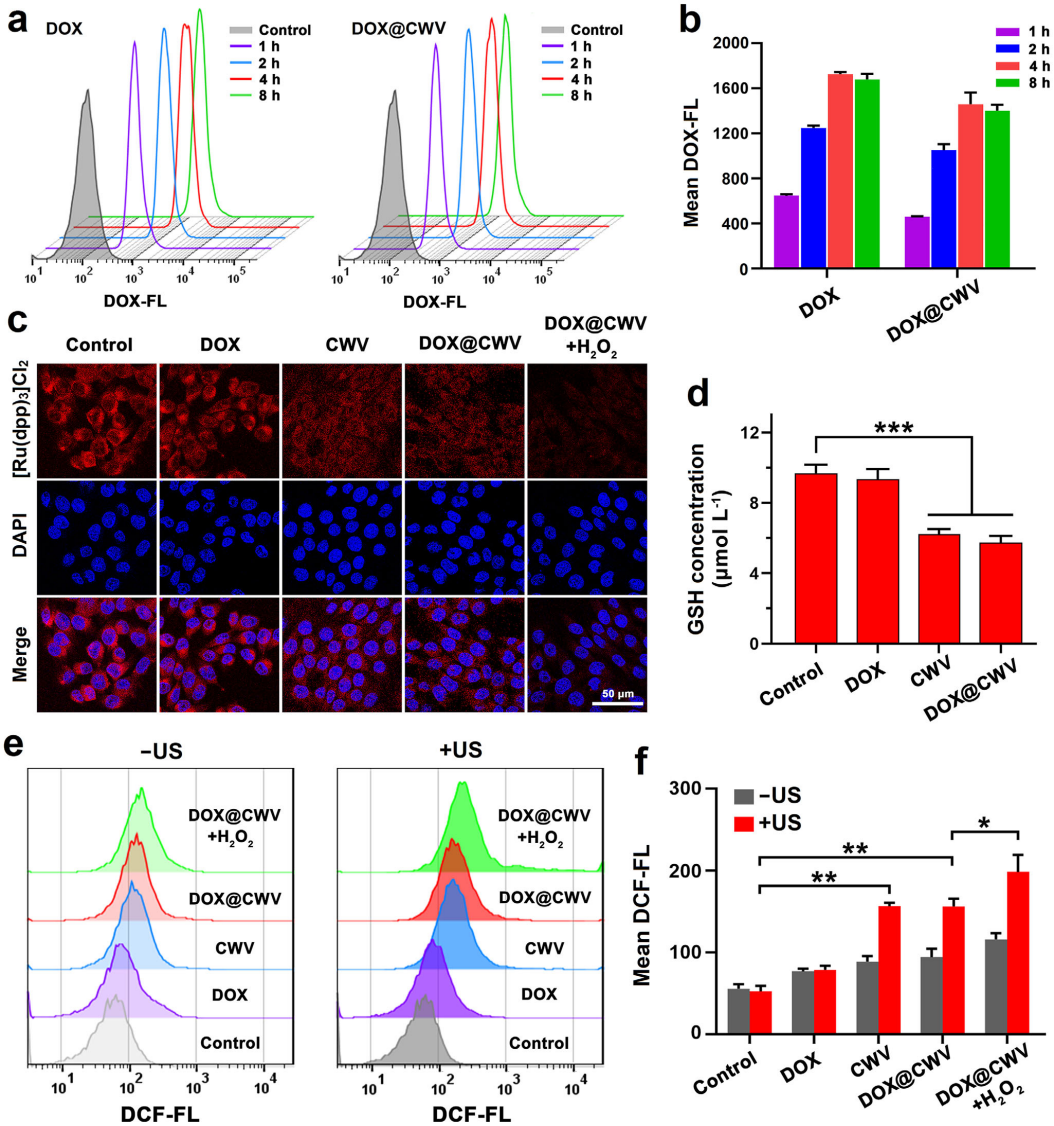
POD‐mimicking and sonodynamic activities of DOX@CWV in cancer cells. a) Flow cytometric plots and b) mean fluorescence intensities (MFI) of DOX in SCC‐7 cells after incubation with free DOX and DOX@CWV for 1, 2, 4, and 8 h. DOX concentration was 1 µg mL^−1^. Data are presented as mean values ± SD (*n* = 3). c) Confocal images of SCC‐7 cells stained with [Ru(dpp)_3_]Cl_2_ after 4‐hour incubation with DOX, CWV, DOX@CWV, and DOX@CWV+H_2_O_2_. Scale bar: 50 µm. d) GSH concentrations in SCC‐7 cells after 4‐hour incubation with DOX, CWV, and DOX@CWV. Data are presented as mean values ± SD (*n* = 5). *** represents *p* < 0.001 between two treatment groups (one‐way ANOVA). e) Flow cytometric plots and f) MFIs of DCF in SCC‐7 cells after various treatments with and without US irradiation. Data are presented as mean values ± SD (*n* = 3). * and ** represent *p* < 0.05 and 0.01 between two treatment groups (one‐way ANOVA). US irradiation (1 MHz, 1.5 W cm^−2^) was performed for 5 min.

The POD‐mimicking activity of DOX@CWV was further evaluated in mouse squamous cell carcinoma (SCC‐7) cells. We first analyzed the cellular uptake of DOX@CWV using flow cytometry. After different times of incubation, DOX and DOX@CWV were efficiently uptaken by SCC‐7 cells at the same DOX concentrations of 1 µg mL^−1^ (**Figure** **3**a) and exhibited no significant differences in their intracellular fluorescence intensities (Figure 3b). It was visible that the cellular uptake of DOX@CWV reached the saturation state after 4 h of incubation. To further confirm the internalization pathway of DOX@CWV, we used endocytosis inhibitors targeting clathrin (chlorpromazine, CPZ), caveolin (filipin III), lipid rafts (methyl‐β‐cyclodextrin, M‐β‐CD), and macropinocytosis (amiloride, AMI). SCC‐7 cell uptake of DOX@CWV was significantly inhibited by CPZ, filipin III, and M‐β‐CD, indicating internalization primarily via clathrin‐, caveolin‐, and lipid raft‐mediated pathways (Figure S3, Supporting Information). Thereby, we further evaluated the POD‐mimicking activity of DOX@CWV in SCC‐7 cells after 4‐hour incubation using a luminescent oxygen sensor [Ru(dpp)_3_]Cl_2_. As shown in the confocal images (Figure 3c), [Ru(dpp)_3_]Cl_2_ displayed obviously weakened fluorescence in SCC‐7 cells incubated with CWV and DOX@CWV, indicating the significant production of intracellular O_2_ induced by these POD‐mimicking catalysts. In SCC‐7 cells cultured with 100 µmol L^−1^ H_2_O_2_, DOX@CWV further promoted the production of intracellular O_2_. Moreover, CWV and DOX@CWV also triggered the reduction of intracellular GSH (Figure 3d). These findings fully demonstrated the potent POD‐mimicking activity of DOX@CWV.

Next, we examined the sonodynamic activity of DOX@CWV in SCC‐7 cells by using DCFH‐DA as a fluorescence probe for detecting the intracellular ROS. The flow cytometry results of intracellular DCF and the comparison of DCF fluorescence intensities are displayed respectively in Figure 3e,f. After 5‐minute US irradiation (1 MHz, 1.5 W cm^−2^), CWV and DOX@CWV both significantly enhanced the levels of intracellular DCF while compared to the control, indicating that these sonosensitizers triggered the generation of large amounts of intracellular ROS. After adding 100 µmol L^−1^ H_2_O_2_ in the culture media, DOX@CWV further elevated the generation level of ROS in SCC‐7 cells, which should be ascribed to their POD‐mimicking activity that catalytically decomposed H_2_O_2_ into O_2_. We also observed the fluorescence of intracellular DCF under a confocal microscope and obtained similar results as described above (Figure S4, Supporting Information). As the mitochondria‐dependent cell damage induced by ROS is one of the main mechanisms of SDT for fighting cancer,^[^ ^33^ ^]^ we next evaluated the changes of mitochondrial membrane potentials in SCC‐7 cells after various treatments using a cationic fluorescence dye, rhodamine 123 (Rh123).^[^ ^34^ ^]^ After US irradiation, DOX@CWV notably boosted the fluorescence of Rh123 in SCC‐7 cells cultured with or without H_2_O_2_, indicating the abnormal mitochondrial membrane potentials caused by the mitochondria damage (Figure S5, Supporting Information). All these results confirmed the excellent sonodynamic activity of DOX@CWV.

### 2.4 In Vitro Anticancer Effects of DOX@CWV‐Mediated Combination Treatment

**Figure 4:**
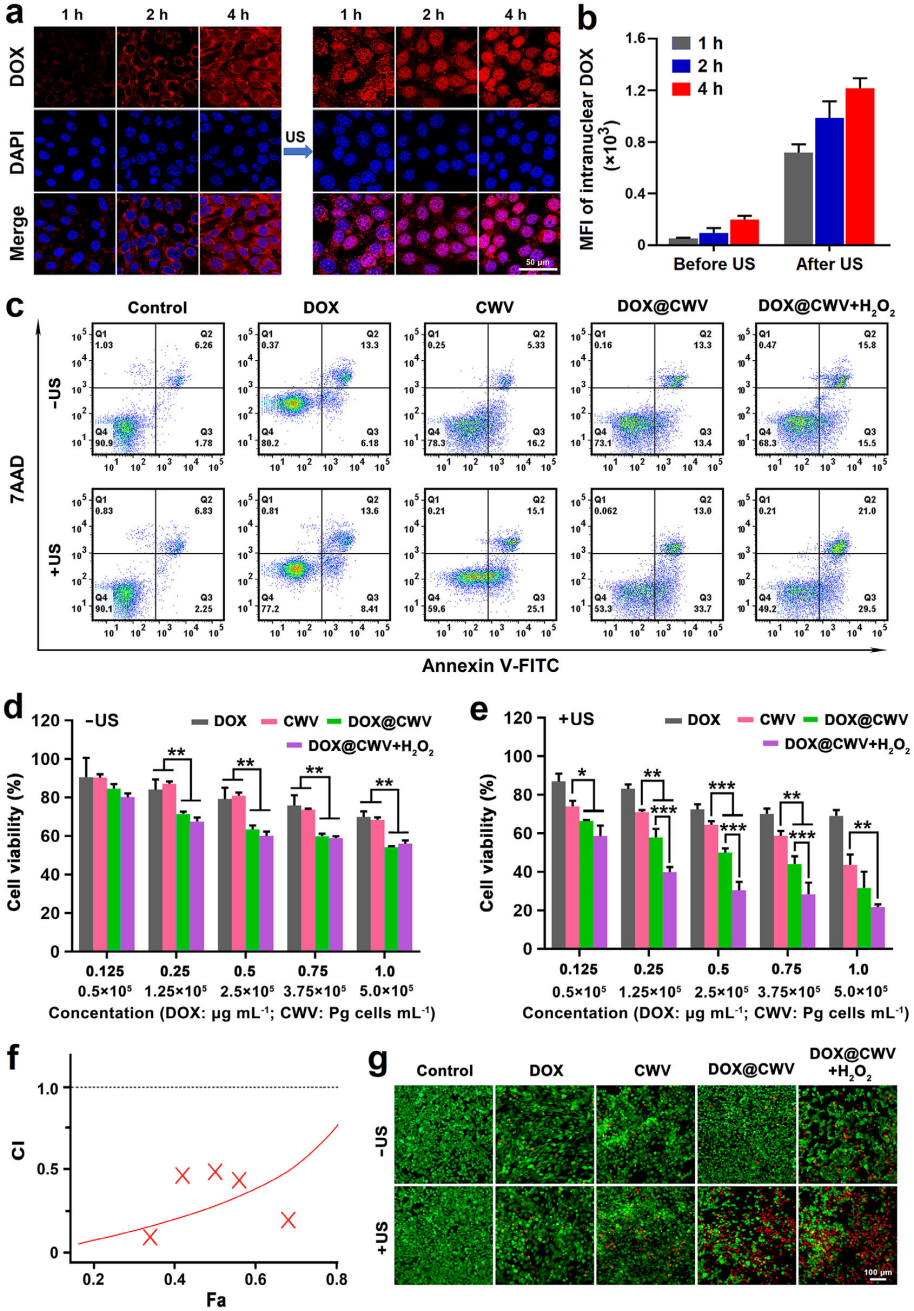
In vitro synergistic anticancer effects of DOX@CWV‐mediated combination treatment. a) Confocal images and b) MFI of intranuclear DOX in SCC‐7 cells while incubation with DOX@CWV before and after US irradiation. Scale bar: 50 µm. c) Flow cytometric plots of apoptosis induction in SCC‐7 cells after the treatments of DOX ± US, CWV ± US, DOX@CWV ± US, and DOX@CWV+H_2_O_2_ ± US. d, e) Cytotoxicities of the above treatments. Data are presented as mean values ± SD (*n* = 5). *, **, and *** represent *p* < 0.05, < 0.01, and < 0.001 between two treatment groups (one‐way ANOVA). f) CI–Fa plots for DOX@CWV‐mediated combination treatment at different concentrations of DOX and CWV. g) Fluorescence microscopic images of Live/Dead stained cells after various treatments. Scale bar: 100 µm. US irradiation (1 MHz, 1.5 W cm^−2^) was performed for 5 min.

Owing to the encapsulation of DOX, DOX@CWV can combine SDT and chemotherapy to synergistically fight and even eliminate cancer cells. However, DOX can exert cytotoxic activity only if it is released from DOX@CWV. Therefore, we observed the intracellular release of DOX from DOX@CWV after incubation and US irradiation. The confocal images of the treated SCC‐7 cells and the fluorescence intensities of intranuclear DOX are shown in **Figure** **4**a,b, respectively. Over a 4‐hour incubation period, the red fluorescence of intracellular DOX sourced from DOX@CWV was elevated gradually, but mostly located in the cytoplasm. It indicated that DOX@CWV was not efficiently released from DOX@CWV after internalization by SCC‐7 cells. However, upon 5‐minute US irradiation (1 MHz, 1.5 W cm^−2^), the fluorescence of DOX began to translocate from the cytoplasm into the cell nuclei and was almost completely located in the cell nuclei after 4 h. This meant that US irradiation accelerated the intracellular release of DOX from DOX@CWV.

Given that ROS generated during SDT can activate the mitochondrial apoptotic pathway^[^ ^35^ ^]^ and DOX can induce the apoptosis of cancer cells through multiple mechanisms,^[^ ^36^ ^]^ we evaluated the synergistic apoptosis‐inducing effect of DOX@CWV‐mediated combination treatment in SCC‐7 cells. The flow cytometry results are given in Figure 4c. While the concentrations of DOX and CWV were 0.5 µg mL^−1^ and 2.5 × 10^5^ Pg cells mL^−1^ respectively, DOX, CWV, and DOX@CWV all induced the cell apoptosis, but DOX@CWV exhibited more potent apoptosis‐inducing effect. After 5‐minute US irradiation (1 MHz, 1.5 W cm^−2^), the apoptosis levels induced by CWV and DOX@CWV were further enhanced obviously, corresponding to the apoptosis rates of 40.2% and 46.7%, respectively. Moreover, DOX@CWV induced the higher rates of apoptotic cells in the culture containing 100 µmol L^−1^ of H_2_O_2_ owing to their POD‐mimicking activity, e.g., the apoptosis rate was increased to 50.5% after US irradiation. These results confirmed the synergistic apoptosis‐inducing effect of DOX@CWV‐mediated combination of SDT and chemotherapy.

Next, MTT assay was used to evaluate the synergistic cytotoxicity of DOX@CWV‐mediated combination treatment in SCC‐7 cells after 48 h. Without US irradiation, DOX and CWV showed only slight cytotoxicity even at the concentrations of DOX and CWV up to 1.0 µg mL^−1^ and 5 × 10^5^ Pg cells mL^−1^, respectively. By comparison, DOX@CWV had significantly stronger cytotoxicity whether with or without the presence of H_2_O_2_, reflecting in the much lower cell viabilities at the same concentrations of DOX and CWV (Figure 4d). CWV at the concentration of 5 × 10^5^ Pg cells mL^−1^ showed no significant cytotoxicity in NIH‐3T3 cells (Figure S6, Supporting Information), indicating that CWV itself possesses high biosafety. As discussed above, US irradiation with a power density of 1.5 W cm^−2^ did not influence the growth of SCC‐7 cells. Here, US irradiation also did not influence the cytotoxicity of DOX, but markedly promoted the cytotoxicity of both CWV and DOX@CWV (Figure 4e), demonstrating the sonodynamic cytotoxicity of these treatments. By comparison, the sonodynamic cytotoxicity of DOX@CWV was much higher than that of CWV. At the same conditions, DOX@CWV exhibited significantly enhanced sonodynamic cytotoxicity in SCC‐7 cells cultured with H_2_O_2_, owing to their own POD‐mimicking activity. The synergistic cytotoxicity of DOX@CWV‐mediated combination treatment was further analyzed by Chou‐Talalay combination index (CI) method.^[^ ^37^ ^]^ As shown in Figure 4f, the CI values at different fraction affected (Fa) were smaller than 1.0, confirming the synergistic cytotoxicity of SDT and chemotherapy based on DOX@CWV. Live/Dead cell staining kit was also used to visually evaluate the cell‐killing effects of the above treatments. Under a fluorescence microscope, the live and dead cells emitted green and red fluorescence, respectively. After US irradiation, DOX@CWV killed the vast majority of SCC‐7 cells cultured with or without H_2_O_2_ (Figure 4g), indicating their potent cell‐killing effect.

### 2.5 In Vitro Specific Anticancer Immune Responses Induced by DOX@CWV‐Mediated Combination Treatment

**Figure 5:**
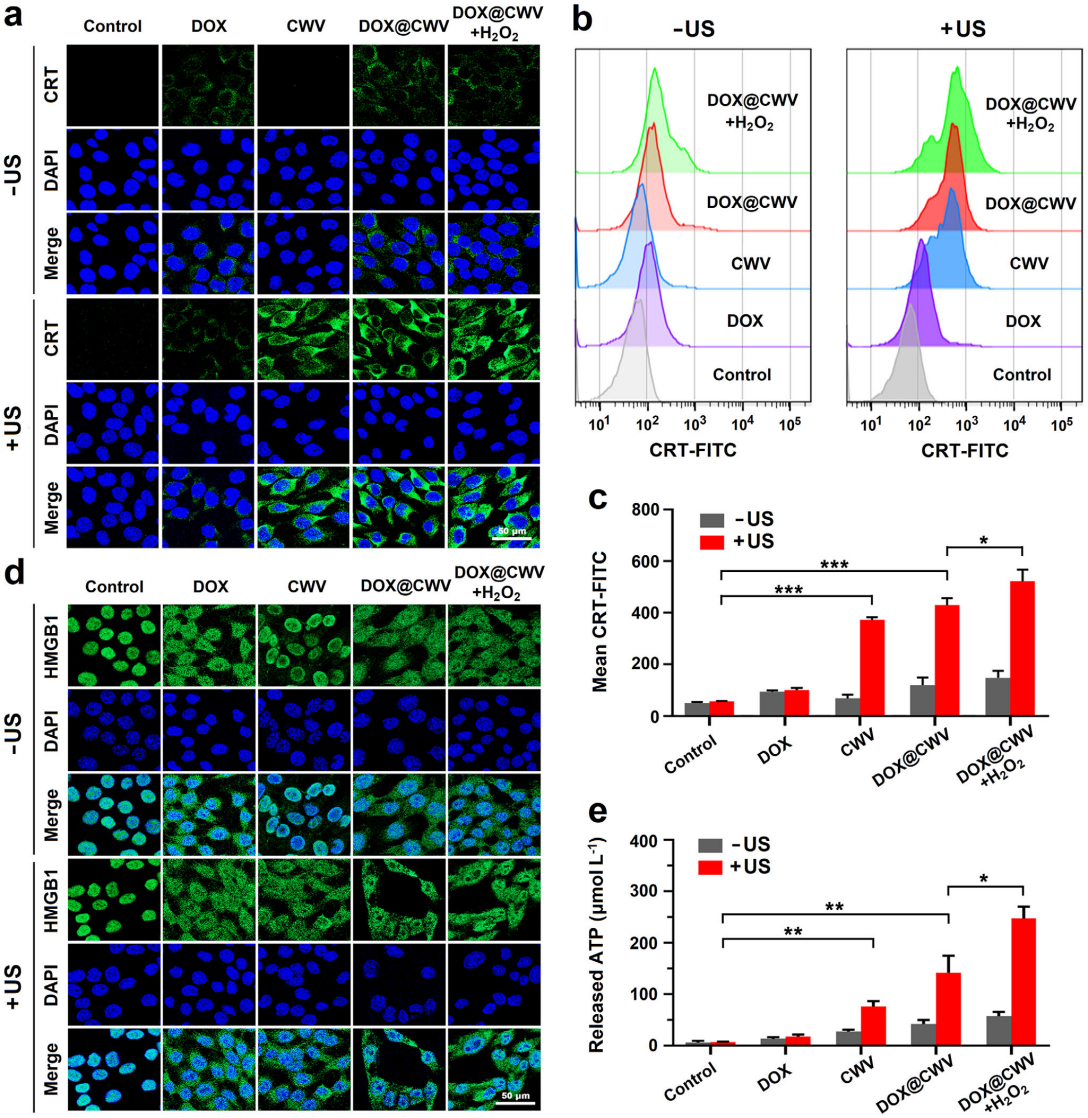
In vitro ICD‐inducing effect of DOX@CWV‐mediated combination treatment. a) Confocal images of SCC‐7 cells with immunofluorescence staining of CRT after the treatments of DOX ± US, CWV ± US, DOX@CWV ± US, and DOX@CWV+H_2_O_2_ ± US. Scale bar: 50 µm. b) Flow cytometry plots and c) MFIs of CRT exposed on the surfaces of SCC‐7 cells after various treatments. d) Confocal images of SCC‐7 cells with immunofluorescence staining of HMGB1 after the treatments of DOX ± US, CWV ± US, DOX@CWV ± US, and DOX@CWV+H_2_O_2_ ± US. Scale bar: 50 µm. e) Quantitative levels of ATP released from SCC‐7 cells after various treatments. Data are presented as mean values ± SD (*n* = 3). *, ** and *** represent *p* < 0.05, 0.01 and < 0.001between two treatment groups (one‐way ANOVA). US irradiation (1 MHz, 1.5 W cm^−2^) was performed for 5 min.

Many investigations have shown that SDT can induce the ICD of cancer cells and thus release DAMPs to stimulate anticancer immune responses.^[^ ^38^, ^39^ ^]^ Herein, we evaluated the ICD‐inducing effects of DOX@CWV‐mediated combination treatment in SCC‐7 cells by detecting the release of DAMPs, including calreticulin (CRT), high‐mobility group box‐1 (HMGB1), and adenosine triphosphate (ATP).^[^ ^40^ ^]^ Immunofluorescence technique was utilized to assess the surface exposure of CRT. After 5‐minute US irradiation (1 MHz, 1.5 W cm^−2^), the fluorescence of CRT became very intensive on the surfaces of SCC‐7 cells treated with CWV and DOX@CWV (**Figure** **5**a). Quantitative analysis was further performed using flow cytometry. The results also demonstrated that CWV and DOX@CWV markedly promoted the surface exposure of CRT after US irradiation (Figure 5b,c). Next, the intracellular localization of HMGB1 was observed under a confocal microscope after immunofluorescence staining. Besides the cells treated with CWV alone, the translocation of HMGB1 from the nucleus to the cytoplasm was clearly observed in all other treated cells (Figure 5d). Finally, the extracellular release of ATP was detected with ATP assay kit. As shown in Figure 5e, CWV and DOX@CWV both significantly promoted the release levels of ATP after US irradiation. In addition, the promotion effects of DOX@CWV with US irradiation on the extracellular release of ATP (Figure 5e) were further enhanced while adding 100 µmol L^−1^ of H_2_O_2_. These results indicated that DOX@CWV‐mediated combination treatment can stimulate the immune responses through inducing the ICD of cancer cells.

**Figure 6:**
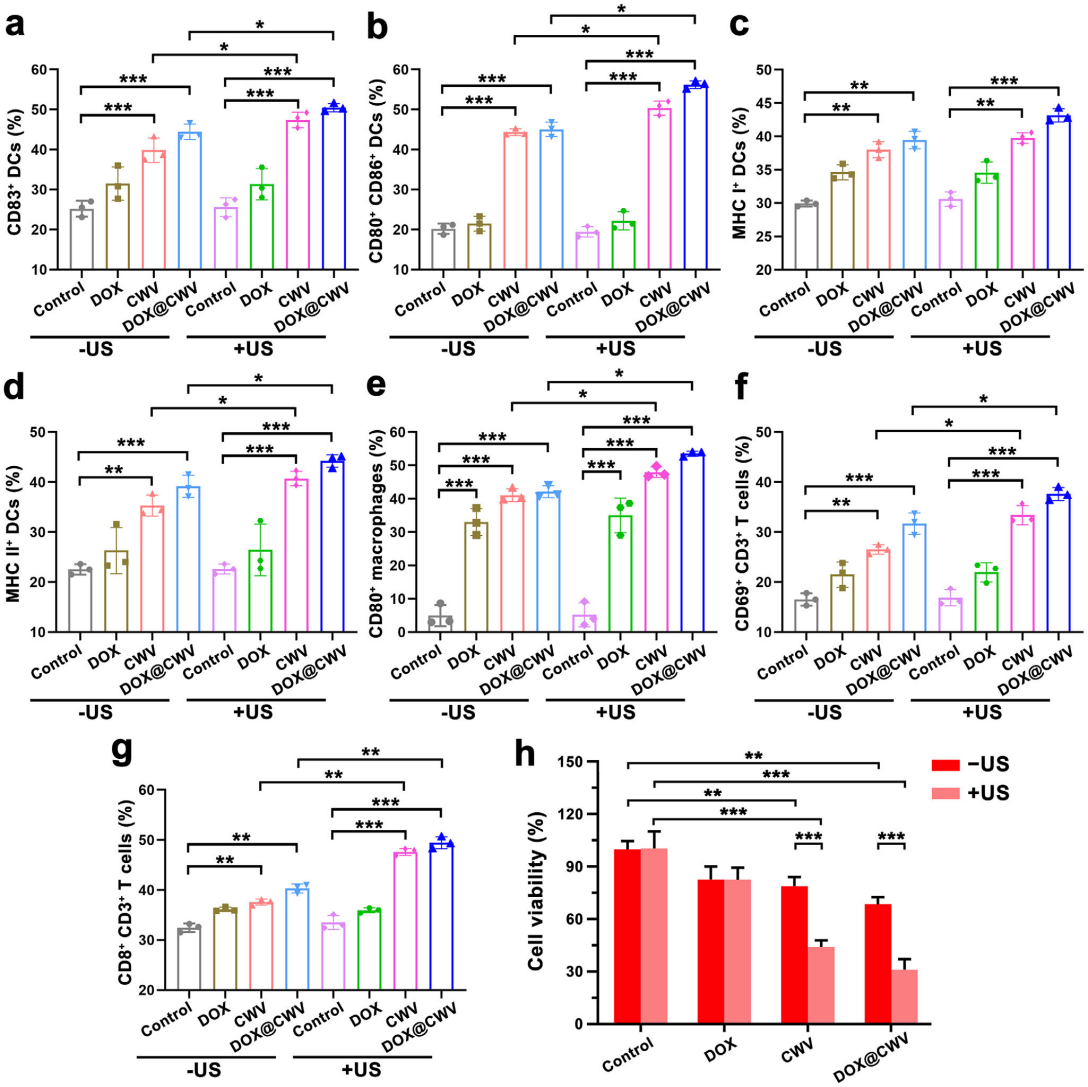
In vitro specific anticancer immune responses of DOX@CWV‐mediated combination treatment. Flow cytometric results of a) CD83^+^ BMDCs, b) CD80^+^ CD86^+^ BMDCs, c) MHC‐I^+^ BMDCs, d) MHC‐II^+^ BMDCs, and CD80^+^ RAW264.7 cells after 48‐hour incubation with the tumoral antigens obtained from SCC‐7 cells receiving the treatments of DOX ± US, CWV ± US, DOX@CWV ± US. e) Flow cytometric results of f) CD69^+^ CD3^+^ early activated T cells and g) CD8^+^ CD3^+^ cytotoxic T cells after 48‐hour incubation with BMDCs and the tumoral antigens obtained from SCC‐7 cells receiving various treatment. Data are presented as mean values ± SD (*n* = 3). *, ** and *** represent *p* < 0.05, 0.01 and < 0.001 between two treatment groups (One‐way ANOVA). h) Viabilities of SCC‐7 cells co‐cultured with BMDCs and splenic T cells after 48‐hour incubation with the tumoral antigens obtained from SCC‐7 cells receiving various treatments. Data are presented as mean values ± SD (*n* = 5). ** and *** represent *p* < 0.01 and < 0.001 between two treatment groups (One‐way ANOVA). US irradiation (1 MHz, 1.5 W cm^−2^) was performed for 5 min.

Owing to the efficient retention of PAMPs such as LPS on the bacterial CWs, DOX@CWV can theoretically stimulate the innate immunity to amplify the anticancer immune responses triggered by the ICD of cancer cells. Herein, we evaluated the anticancer immune responses stimulated by DOX@CWV‐mediated combination treatment, including the activation and maturation of DCs, the differentiation of effector T cells, and the polarization of M1 macrophages. First, bone marrow‐derived DCs (BMDCs) were isolated from healthy C57BL/6 mice and then incubated with the tumoral antigens obtained from SCC‐7 cells receiving various treatments as mentioned above. After incubation for 48 h, BMDCs were processed with immunofluorescence staining and their surface biomarkers were next analyzed with a flow cytometer according to our previous report.^[^ ^41^ ^]^ Whether with or without US irradiation, CWV and DOX@CWV both greatly upregulated the expressions of CD83, a marker molecule for mature DCs (**Figure** **6**a; Figure S7, Supporting Information), co‐stimulatory molecules of CD80/CD86 (Figure 6b; Figure S8, Supporting Information), major histocompatibility complex class I (MHC‐I) (Figure 6c; Figure S9, Supporting Information), and MHC class II (MHC‐II) (Figure 6d; Figure S10, Supporting Information), demonstrating that all these treatments exerted strong promotion effects on the activation of BMDCs. As LPS on the surfaces of DOX@CWV can be recognized by PRRs and subsequently promote the polarization of M1 macrophages,^[^ ^42^ ^]^ we next analyzed the M1 specific marker of CD80 on mouse macrophage RAW264.7 cells after various treatments. As shown in the results of flow cytometry, all treatments dramatically boosted the expression levels of CD80 on RAW264.7 cells (Figure 6e; Figure S11, Supporting Information), confirming their strong inducing effects on the M1 polarization. By comparison, CWV and DOX@CWV showed more potent effects on the activation of these immune cells after US irradiation, reflecting in the significantly upregulated CD80/CD86 on BMDCs (Figure 6b) and CD80 on RAW264.7 cells (Figure 6e). It thus can be deduced that DAMPs released from dying cancer cells after SDT and bacterial PAMPs synergistically exerted immune activation effects.

Mature DCs can present cancer antigens to T cells and subsequently promote their differentiation into specific effector T cells, which in turn stimulate specific immune responses to fight cancer cells. We used the co‐culture system of BMDCs, T cells isolated from the spleens of healthy C57BL/6 mice, and SCC‐7 cells for investigating the specific anticancer immune responses. After 48‐hour incubation with the tumoral antigens obtained from SCC‐7 cells receiving various treatments, the surface markers of T cells were analyzed using flow cytometry after immunofluorescence staining. Whether with or without US irradiation, CWV and DOX@CWV both notably upregulated the expression levels of CD69 (an early activation marker^[^ ^43^ ^]^) on CD3^+^ T cells (Figure 6f; Figure S12, Supporting Information) and also significantly enhanced the proportions of cytotoxic CD8^+^ CD3^+^ T cells (Figure 6g; Figure S13, Supporting Information), confirming the activation and differentiation of T cells promoted by these treatments. By comparison, CWV and DOX@CWV exhibited more potent promotion effects on T cells after US irradiation. It can be seen that SDT and bacterial immunotherapy synergistically exerted immune stimulation effects. The culture media were collected from the above co‐culture systems for examining the secretion levels of pro‐inflammatory cytokines, including tumor necrosis factor‐α (TNF‐α), interferon‐γ (IFN‐γ), and interleukin‐12p70 (IL‐12p70) that have potent anticancer activity according to the previous reports.^[^ ^44^ ^]^ As shown in Figure S14 (Supporting Information), the secretion levels of TNF‐α, IFN‐γ, and IL‐12p70 determined by ELISA assay were consistent with the above results of the activation of T cells. DOX@CWV with US irradiation exhibited the most potent promoting effects on the secretion levels of these cytokines. Given T cells' role in immunity, assessing CWV's effects is key to its safety profile. Cytotoxicity tests (Figure S15, Supporting Information) showed CWV had no significant impact on T cell viability across tested concentrations. Finally, MTT assay was used to evaluate the viabilities of SCC‐7 cells in the above co‐culture systems. All treatments inhibited the growth of SCC‐7 cells at different levels, but the inhibitory effects of CWV and DOX@CWV were markedly enhanced after US irradiation. Only about 30% of SCC‐7 cells were survived in the treatment group of DOX@CWV with US irradiation (Figure 6h), demonstrating that DOX@CWV‐mediated combination treatment stimulated the specific anticancer immunity.

### 2.6 In Vivo Anticancer Effects of DOX@CWV‐Mediated Combination Treatment

**Figure 7:**
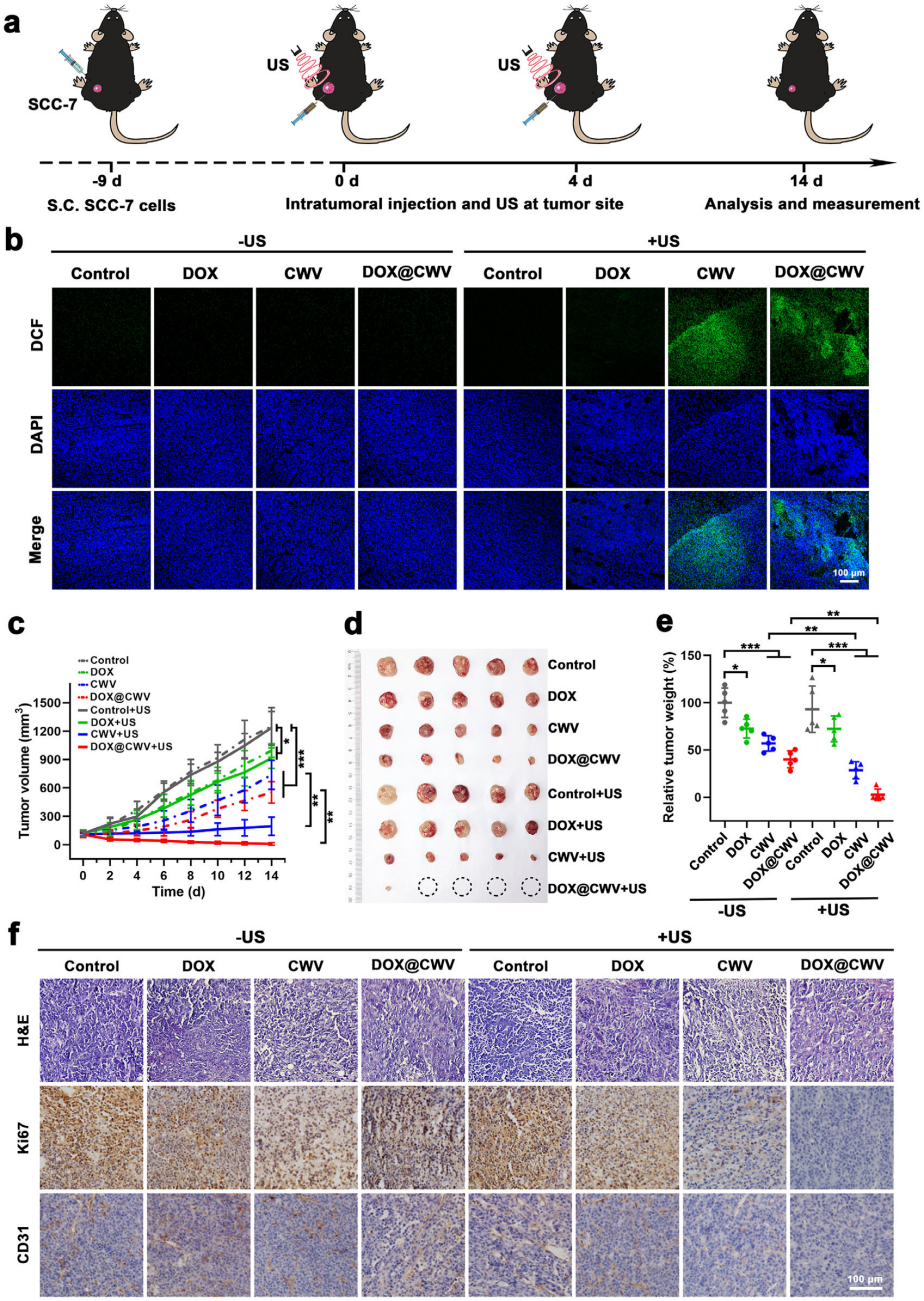
Synergistic anticancer effects of DOX@CWV‐mediated combination treatment in SCC‐7 tumor‐bearing mice. a) Schematic illustration for therapeutic schedule. b) Confocal images of tumor sections processed with DCFH‐DA from the mice receiving the treatments of PBS ± US (the control ± US), DOX ± US, CWV ± US, and DOX@CWV ± US. Scale bar: 100 µm. c) Growth curves of the tumors within 14 d from the beginning of treatments. d) Photograph and e) weights of the isolated tumors. Data are presented as mean values ± SD (*n* = 5). *, ** and *** represent *p* < 0.05, 0.01, and < 0.001 between two treatment groups (One‐way ANOVA). f) Microscope images of tumor sections with H&E staining and immunohistochemical staining of Ki67 and CD31. Scale bar: 100 µm. In the above experiments, the mice were administered through intratumoral injection at the DOX and CW doses of 0.15 mg kg^−1^ and 7.5 × 10^7^ Pg cells kg^−1^, respectively. US irradiation (1 MHz, 1.5 W cm^−2^) was performed at the tumor site for 5 min.

An animal model of oral cancer was established by subcutaneously inoculating SCC‐7 cells into the outside hind legs of C57BL/6 mice for evaluating the in vivo anticancer effects of DOX@CWV‐mediated combination treatment. Treatments including DOX, CWV, and DOX@CWV with or without US irradiation were carried out according to the therapeutic schedule in **Figure** **7**a. The mice were administrated with PBS (the control), DOX, CWV, and DOX@CWV through intratumoral injection. The doses of DOX and CWV were 0.15 mg kg^−1^ and 7.5 × 10^7^ Pg cells kg^−1^, respectively. After 4 h, these mice received 5‐minute US irradiation (1 MHz, 1.5 W cm^−2^) at the tumor site. For evaluating sonodynamic activity, DCFH‐DA was used as a fluorescent probe to detect the generation of intratumoral ROS. Figure 7b and Figure S16 (Supporting Information) show the fluorescence microscopic images and semi‐quantitative analysis of tumor frozen sections processed with DCFH‐DA. Only upon US irradiation, the tumors treated with CWV and DOX@CWV displayed the intensive green fluorescence of DCF, showing the generation of intratumoral ROS triggered by these treatments. These results suggest that CWV and DOX@CWV are likely to be excellent sonosensitizers for cancer SDT.

SCC‐7 tumor‐bearing mice were given the above treatments twice respectively on the 0th and 4th day, and the tumor volumes and the body weights were measured continuously for 14 d from the beginning of treatments. Afterwards, all the mice were sacrificed and their tumors, major organs, and blood samples were isolated for further examinations. Tumor growth curves are shown in Figure 7c and Figure S17 (Supporting Information). Whether with or without US irradiation, DOX, CWV, and DOX@CWV all notably inhibited tumor growth, but CWV and DOX@CWV showed much more potent inhibitory effects than DOX. Upon US irradiation, the inhibitory effects of CWV and DOX@CWV were both further enhanced significantly, especially for DOX@CWV which almost completely inhibited tumor growth. The mice receiving the treatment of DOX@CWV with US irradiation displayed the smallest tumor size (Figure 7d) and the lowest tumor weights (Figure 7e), and only one mouse had tumor recurrence. The tumors collected from the treated mice were embedded in paraffin and cut into 5 µm‐thick sections. Hematoxylin and eosin (H&E) staining was performed on these tumor sections for observing the morphological changes of tumor cells. At the same time, the immunohistochemical staining of Ki67 (a proliferation marker^[^ ^45^ ^]^) and CD31 (a vascular endothelial marker^[^ ^46^ ^]^) were carried out to examine the proliferation of tumor cells and tumor angiogenesis, respectively. DOX@CWV with US irradiation also exhibited the strongest anticancer effects. In this treatment group, tumor cells displayed necrosis, irregular shapes, deformed, shrunk or even dissolved nuclei, and markedly inhibited proliferation, and meanwhile, tumor angiogenesis was notably suppressed (Figure 7f). Compared to the control mice, all the treated mice showed no visible decrease in their body weights (Figure S18, Supporting Information), no discernible histopathological injuries in their major organs (Figure S19, Supporting Information), and no significant changes in their routine blood test and biochemical analysis data (Figure S20, Supporting Information), preliminarily confirming the biosafety of these treatments.

### 2.7 In Vivo Antimetastatic Effects of DOX@CWV‐Mediated Combination Treatment

**Figure 8:**
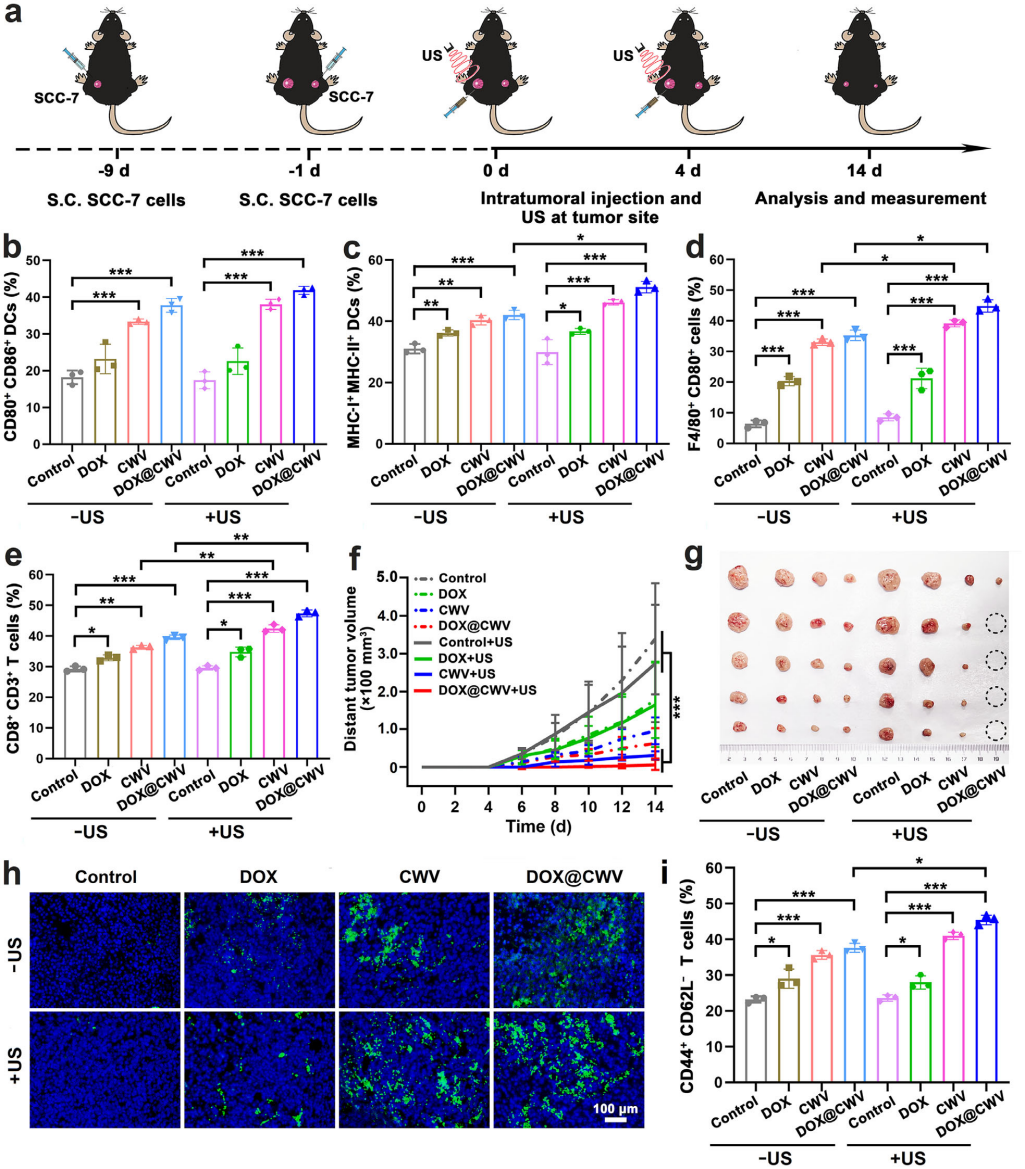
Immunostimulatory and antimetastatic effects of DOX@CWV‐mediated combination treatment in bilateral SCC‐7 tumor‐bearing mice. a) Schematic illustration of therapeutic schedule. Flow cytometric results of b) CD80^+^ CD86^+^ DCs, c) MHC‐I^+^/MHC‐II^+^ DCs, d) CD80^+^ M1 macrophages gated on F4/80, e) CD8^+^ CD3^+^ cytotoxic T cells in tumor tissues isolated from the mice receiving the treatments of PBS ± US (the control ± US), DOX ± US, CWV ± US, and DOX@CWV ± US. Data are presented as mean values ± SD (*n* = 3). *, **, and *** represent *p* < 0.05, < 0.01, and < 0.001 between two treatment groups (One‐way ANOVA). f) Growth curves of distant tumors in the mice receiving various treatments. Data are presented as mean values ± SD (*n* = 5). *** represent *p* < 0.001 between two treatment groups (One‐way ANOVA). g) Photograph of distant tumors isolated from the mice after various treatments. h) Fluorescence microscopic images of CD8 immunofluorescence‐stained sections of distant tumors. Scale bar: 100 µm. i) Flow cytometric plots of CD44^+^ CD62^–^ splenic T cells isolated from the mice after various treatments. Data are presented as mean values ± SD (*n* = 3). * and *** represent *p* < 0.05 and < 0.001 between two treatment groups (One‐way ANOVA). In the above experiments, the mice were administered with sample solutions through intratumoral injection at the DOX and CW doses of 0.15 mg kg^−1^ and 7.5 × 10^7^ Pg cells kg^−1^, respectively. US irradiation (1 MHz, 1.5 W cm^−2^) was performed at the primary tumor site for 5 min.

An animal model of metastatic oral cancer was established to evaluate the antimetastatic effects of DOX@CWV‐mediated combination treatment. SCC‐7 cells were inoculated into the left outside hind legs of C57BL/6 mice through subcutaneous injection as the primary tumors and further inoculated into the right outside hind legs of these mice after 8 d as the distant tumors. These mice were then given the treatments as mentioned above according to the schedule illustrated in **Figure** **8**a. In view of the fact that immune stimulation can contribute to cancer metastasis suppression,^[^ ^47^ ^]^ we analyzed the stimulation of immune responses in the treated mice. At 2 d after the first treatments, 3 mice were selected randomly from each group, and their tumors and spleens were isolated for examining the activation of immune cells using flow cytometry. Whether with or without US irradiation, CWV and DOX@CWV markedly elevated the proportions of CD80^+^ CD86^+^ DCs (Figure 8b; Figure S21, Supporting Information), MHC‐I^+^ MHC‐II^+^ DCs (Figure 8c; Figure S22, Supporting Information), F4/80^+^ CD80^+^ macrophages (Figure 8d; Figure S23, Supporting Information), and CD8^+^ CD3^+^ T cells (Figure 8e; Figure S24, Supporting Information), demonstrating that these treatments successfully stimulated multiple immune cells in the TME. However, the immunostimulatory effects of CWV and DOX@CWV with US irradiation were notably more robust than those without US irradiation. Similar trends were observed in the flow cytometric results of spleen tissues, reflecting in the enhanced proportions of CD80^+^ CD86^+^ DCs (Figure S25, Supporting Information) and MHC‐I^+^ MHC‐II^+^ DCs (Figure S26, Supporting Information), as well as the reduced proportions of CD4^+^ CD25^+^ Foxp3^+^ regulatory T cells (Figure S27, Supporting Information). These findings confirmed that CWV and DOX@CWV exerted the synergistic immunostimulatory effects of bacterial immunotherapy and SDT against cancer.

Within 14 d after the beginning of treatments, the volumes of the distant tumors were measured continuously. As shown in Figure 8f and Figure S28 (Supporting Information), CWV and DOX@CWV both significantly suppressed the growth of distant tumors whether with or without US irradiation, but they exhibited much stronger inhibitory effects after US irradiation. After that, the mice were sacrificed, and their tumors and spleens were isolated for the following examinations. DOX@CWV with US irradiation almost completely suppressed the growth of distant tumors and only one mouse showed distant tumor (Figure 8g). Next, the tumors were frozen sectioned and processed with CD8 immunofluorescence staining to observe the infiltration of cytotoxic T cells. The confocal images showed that CWV and DOX@CWV both visibly promoted the infiltration of cytotoxic CD8^+^ T cells in the TME whether with or without US irradiation (Figure 8h; Figure S29, Supporting Information). The spleens were further homogenized for analyzing the differentiation of effector memory T cells using flow cytometry, and the results are displayed in Figure 8i and Figure S30 (Supporting Information). Compared to the control, all treatments significantly elevated the proportions of CD44^+^ CD62L^−^ memory T cells, confirming the generation of long‐lasting immunological memory. DOX@CWV with US irradiation also exerted the most potent promotion effects on the above immune responses. These findings suggest that DOX@CWV‐mediated combination treatment can suppress cancer metastasis by overcoming the immunosuppressive TEM and activating systematic immunity.

## 3 Conclusions

In this study, bacterial CWs with intrinsic POD‐mimicking and sonodynamic activities were extracted from Pg and further nanofabricated for encapsulating the chemotherapeutic agent DOX. DOX@CWV thus obtained had very potent sonodynamic performance against cancer cells owing to their POD‐mimicking activity. Our results revealed that DOX@CWV efficiently inhibited the growth of primary cancer through combining SDT and chemotherapy, and also exerted the synergistic immunostimulatory effects of bacterial immunotherapy and SDT to suppress the distant metastasis. The DOX@CWV‐mediated combination treatment demonstrates a promising and effective strategy for cancer eradication. The biosafety of DOX@CWV was evaluated preliminarily in this study, whereas their possible adverse effects, especially the immunotoxicity sourced from bacterial CWs, require more attention. In the future work, we will comprehensively and thoroughly investigate the in vivo biosafety of DOX@CWV and thus promote their clinical application.

## 4 Experimental Section

### Materials

DOX·HCl and filipin III were purchased from Meilun Biotech (Dalian, China). Lysozyme and CPZ were obtained from Solarbio Science & Technology (Beijing, China). ToxinSensor™ Chromogenic LAL Endotoxin Assay Kit was purchased from GenScript (New Jersey, USA). M‐β‐CD was obtained from MedChemExpress (New Jersey, USA). AMI was purchased from Aladdin (Shanghai, China). DCFH‐DA, DPBF, TMB, and GSH were all bought from Sigma‐Aldrich (St Louis, MO, USA).

### Cell Lines and Animals

The bacterial strain Pg (code BNCC337441) was sourced from BeNa Culture Collection (Beijing, China) and cultured in sheep blood agar plates or Brain Heart Infusion (BHI) broth incorporating 0.5% chlorhematin and 0.5% vitamin K1 in an anaerobic chamber with an atmosphere of 80% N_2_, 10% H_2_, and 10% CO_2_ at 37 °C. Mouse squamous cell cancer SCC‐7 cell line was sourced from OTWO Biotech (Shenzhen, China) and cultured in RPMI 1640 medium (Meilun Biotech, Dalian, China). Mouse macrophage RAW 264.7 cell line and mouse embryonic cell line NIH‐3T3 were sourced from BioVector NTCC (Beijing, China) and cultured in Dulbecco's modified Eagle's medium (DMEM, Meilun Biotech, Dalian, China). The above cell culture media were added with 10% FBS and 1% penicillin/streptomycin and cultured in a humidified atmosphere containing 5% CO_2_ at 37 °C. For SDT treatment, the cells were incubated with therapeutic agents and received 5‐minute US irradiation (1 MHz, 1.5 W cm^−2^) after 4 h using an Intelect® ultrasonic apparatus (chattanooga, USA). The detailed cell experiments were described in the Supporting Information.

Female C57BL/6 mice with 4‒6 weeks old were provided by SPF Biotechnology (Beijing, China). For evaluating the inhibitory effects of DOX@CWV‐mediated combination treatment against oral cancer, a tumor mouse model was established by subcutaneously inoculating 5 × 10^5^ SCC‐7 cells into the outside hind legs of the mice. These tumor‐bearing mice were administrated through intratumoral injection of therapeutic agents and some of them were further received 5‐minute US irradiation (1 MHz, 1.5 W cm^−2^) with an ultrasonic apparatus. All treatments were performed at the tumor site every 4 d for a total of 2 times. A bilateral tumor mouse model was constructed for further evaluating the antimetastatic effects of DOX@CWV‐mediated combination treatment. Briefly, the mice were first inoculated with 5 × 10^5^ SCC‐7 cells at the left outside hind legs as the primary tumors, and 8 d later further inoculated with 5 × 10^5^ SCC‐7 cells at the right outside hind legs as the distant tumors. These mice received the same treatments as mentioned above at the primary tumor site. All animal experiments were performed in accordance with the guidelines of the Ethics Committee of Tianjin Medical University (TMUaMEC2022032). The detailed animal experiments were given in the Supporting Information.

### Preparation and Characterization of DOX@CWV

Bacterial CWs were extracted from Pg cells using the method reported previously^[^ ^24^ ^]^ with some modifications. Briefly, Pg colonies were picked up from sheep blood plates and washed 3 times with PBS. Bacterial cells were collected from 1 × 10^4^ CFU Pg colonies by centrifugating at 3000 g for 10 min at 4 °C, and subsequently resuspended in 20 mL of pH 8.0 Tris‐HCl buffer containing 1 mol L^−1^ sucrose. These bacterial cells were processed with 2 mg mL^−1^ lysozyme under stirring at 120 rpm for 3 h and then centrifuged at 3000 g for 10 min at 4 °C. The black brown supernatants were collected and further passed through a 50 kDa MWCO centrifugal ultrafilter (Merck KGaA, Darmstadt, Germany), thus removed lysozyme to obtain bacterial CWs. EDS elemental mapping was performed to examine the elemental composition of bacterial CWs using a high‐resolution TEM (HR‐TEM, JEM 2100plus, JEOL, Japan), and the content of Fe was measured with an ICP‐MS spectrometer (Agilent 7700, USA).

Bacterial CWs obtained from 1.5 × 10^7^ Pg cells were resuspended in 1 mL deionized water, sonicated in an ice bath at 100 W for 5 min, and then extruded through a 200 nm polycarbonate membrane‐installed mini‐extruder (Avanti Polar Lipid, USA) to prepare CWV. The UV–vis absorption spectra of CWV at concentration of 5 × 10^5^ Pg cells mL^−1^ as well as free heme with a concentration of 10 µg mL ^−1^ were recorded using an UV–vis spectrophotometer (U‐3310, Hitachi, Japan). Next, CWV were incubated with different amounts of DOX (7.5‒150 µg) under stirring for 4 h and further processed by ultrafiltration using a 3 kDa MWCO centrifugal ultrafilter (Merck KGaA, Darmstadt, Germany). DOX@CWV was obtained from the concentrated solutions, and meanwhile the filtrates were collected for detecting the unencapsulated DOX using a F7000 fluorescence spectrophotometer (Hitachi, Japan). The encapsulation efficiencies of DOX were calculated accordingly. DOX@CWV prepared from 30 µg of DOX and CWV fabricated from 1.5 × 10^7^ Pg cells were used in the following experiments.

CWV and DOX@CWV were morphologically characterized with a TEM (HT7700, Hitachi, Japan) after negative staining with 2% phosphotungstic acid. The particle diameters and PDIs of CWV and DOX@CWV were detected using a Zeta‐Sizer detector (Nano ZS90, Malvern, England). The UV‐vis absorption spectra of heme, DOX, CWV, and DOX@CWV were recorded using an UV‐vis spectrophotometer. The colloidal stabilities of DOX@CWV in deionized water, PBS, and 10% FBS were measured by monitoring the changes of their particle sizes and PDIs within 10 d of storage at 4 °C.

The LPS contents of Pg cells, CWV, and DOX@CWV were determined with a LAL endotoxin assay kit according to the manufacture's instruction. SDS‐PAGE was further employed to evaluate the protein components. Protein samples were extracted from Pg cells, protoplasts, CWs, CWV, and DOX@CWV, and further quantified with a BCA protein assay kit (Meilun Biology Technology, Dalian, China). These samples containing 30 µg of proteins were separated by 12% SDS‐PAGE electrophoresis and next stained with Coomassie Brilliant Blue. Protein bands thus obtained were photographed for comparison.

### In Vitro Drug Release of DOX@CWV

The release profiles of DOX from DOX@CWV were evaluated under different conditions using dynamic dialysis method. DOX@CWV containing 30 µg DOX was preprocessed with 5‐minute US irradiation (1 MHz, 1.5 W cm^−2^). Next, DOX@CWV with and without US irradiation were transferred into the dialysis bags with the MWCO of 2 kDa and then dialyzed separately in PBS solutions with different pH values under shaking at 100 rpm at 37 °C. At the scheduled times, the release media were taken out and the fresh release media were added meanwhile. Subsequently, the released amounts of DOX were detected using a fluorescence spectrophotometer.

### POD‐Mimicking Activity of DOX@CWV in Solution

The H_2_O_2_ decomposition activities of CWV and DOX@CWV were evaluated by detecting the production of O_2_ in 100 µmol L^−1^ H_2_O_2_ solutions using a dissolved O_2_ meter (JPBJ‐608, Shanghai, China). Here, the concentrations of DOX and CWV were 10 µg mL^−1^ and 5 × 10^6^ Pg cells mL^−1^, respectively. The O_2_ production levels in the solutions were recorded every 30 s.

The GSH consumption capability of DOX@CWV was evaluated through detecting the concentrations of thiols using Ellman's reagent (MedChemExpress, New Jersey, USA). DOX@CWV with different concentrations of CWV were reacted with 0.25 mmol L^−1^ GSH in PBS for 2, 4, 6, and 12 h. Next, the reactant solutions were collected, centrifuged, and further processed with DTNB at a concentration of 0.05 mmol L^−1^. Subsequently, the concentrations of thiols were determined according to the manufacture's protocol.

### Sonodynamic Activity of DOX@CWV

DCFH‐DA was dissolved in 10 mmol L^−1^ NaOH solution containing the appropriate amount of dimethyl sulfoxide (DMSO), and further reacted for 30 min in the dark. PBS was added to terminate the reaction and thus obtained DCFH at a concentration of 40 µmol L^−1^. Next, DCFH was added separately into the solutions of DOX, CWV, and DOX@CWV supplemented with or without 100 µmol L^−1^ H_2_O_2_. Here, the concentrations of DOX and CWV were 3 µg mL^−1^ and 1.5 × 10^6^ Pg cells mL^−1^, respectively. The mixture solutions were processed with 5‐minute US irradiation (1 MHz, 1.5 W cm^−2^), and the produced DCF were subsequently detected using a fluorescence spectrophotometer. The generation levels of ROS triggered by DOX@CWV upon US irradiation at different power intensities (0, 0.5, 1.0, 1.5, 2.0, and 2.5 W cm^−2^), different concentrations of CWV (0, 0.5, 1.0, 1.5, 2.0, and 2.5 × 10^6^ Pg cells mL^−1^), and different times (0, 1, 2, 3, 4, and 5 min) were also examined at the same time.

DPBF was used as an indicator to detect the generation of ^1^O_2_ triggered by DOX@CWV. Briefly, DOX@CWV with different concentrations of CWV (0, 0.5, 1.0, 1.5, 2.0, and 2.5 × 10^6^ Pg cells mL^−1^) were mixed with 40 µL of DPBF solution (1 mg mL^−1^ in DMSO), and then processed with 5‐minute US irradiation (1 MHz, 1.5 W cm^−2^). Subsequently, the absorbances of DPBF at 415 nm were determined using a UV‐vis spectrophotometer. By using the same method, we further evaluated the influence of US irradiation time (0‒5 min) on the generation of ^1^O_2_ triggered by DOX@CWV.

TMB was used to evaluate the generation of ·OH triggered by DOX@CWV. Briefly, DOX@CWV with different concentrations of CWV (0, 0.5, 1.0, 1.5, 2.0, and 2.5 × 10^6^ Pg cells mL^−1^) were mixed with 40 µL of TMB solution (1 mg mL^−1^ in NaAc/HAc buffer, pH 5.5), and next processed with 5‐minute US irradiation (1 MHz, 1.5 W cm^−2^). After incubation at 37 °C for 30 min, the UV‐vis spectra of TMB in these solutions were recorded using an UV‐vis spectrophotometer.

### Statistical Analysis

All data were from at least three independent experiments and presented as mean ± standard deviation (SD). Statistical comparisons were performed by one‐way ANOVA with Tukey's test. Statistical significance was performed at *p* < 0.05.

## Conflict of Interest

The authors declare no conflict of interest.

## Author Contributions

M.Y. and Y.M. contributed equally to this work. Y.W., Y.L. (Yang Liu), and X.Y. conceived the conceptual ideas and designed the study. M.Y., Y.M., J.L., L.B., and Y.C. performed the experiments. M.Y., Y.M., J.L., Y.L. (Yang Liu), Y.L. (Yuanyuan Liu), and M.C. analyzed the data. Y.W., M.Y., and Y.L. (Yang Liu) wrote the manuscript. Y.L. (Yang Liu), Y.W., and X.Y. supervised the study. All the authors contributed to the manuscript.

## Supporting information



Supporting Information

## Data Availability

The data that support the findings of this study are available from the corresponding author upon reasonable request.
