# Supplementary material for: Nanofabricated Bacterial Cell Walls with Intrinsic Peroxidase‐Mimicking and Sonodynamic Activities for Cancer Combination Treatment
Source: Adv Sci (Weinh). 2025 May 14;12(30):2505310. doi: 10.1002/advs.202505310 (PMC12376657; doi:10.1002/advs.202505310)
Supplement: Supplementary file 1 — Supporting Information [file ADVS-12-2505310-s001.docx]

**Supporting Information**

**Nanofabricated Bacterial Cell Walls with** **Intrinsic Peroxidase-Mimicking and Sonodynamic Activities for** **Cancer Combination Treatment**

*Meng Yang ^1,^ †, Yang Meng ^1,^ †, Junling Li ^3^, Liya Bai ^1^, Yuanyuan Cheng ^2^, Yuanyuan Liu ^3^, Mingxin Cao ^4^, Xiaoying Yang ^1,^ *, Yinsong Wang ^1,4,^ *, and Yang Liu^5,6,^ **

AUTHOR INFORMATION

^1^ Key Laboratory of Immune Microenvironment and Disease (Ministry of Education), The Province and Ministry Co-sponsored Collaborative Innovation Center for Medical Epigenetics, International Joint Laboratory of Ocular Diseases (Ministry of Education), Tianjin Key Laboratory on Technologies Enabling Development of Clinical Therapeutics and Diagnostics (Theranostics), School of Pharmacy, Tianjin Medical University, Tianjin 300070, China

^2^ Department of Pharmacology, Tianjin Key Laboratory of Inflammatory Biology, Center for Cardiovascular Diseases, Key Laboratory of Immune Microenvironment and Disease (Ministry of Education), The Province and Ministry Co-sponsored Collaborative Innovation Center for Medical Epigenetics, Tianjin Medical University, Tianjin 300070, China

^3^ Department of Genetics, School of Basic Medical Sciences, Tianjin Medical University, Tianjin 300070, China

^4^ Department of Orthodontics, Tianjin Medical University School and Hospital of Stomatology, Tianjin Key Laboratory of Oral Soft and Hard Tissues Restoration and Regeneration, and Institute of Stomatology, Tianjin 300070, China

^5^ Department of Hepatobiliary Cancer, Liver Cancer Center, Tianjin Medical University Cancer Institute & Hospital, National Clinical Research Center for Cancer, Key Laboratory of Cancer Prevention and Therapy, Tianjin’s Clinical Research Center for Cancer, Tianjin 300060, China

^6^ Department of Hepatobiliary and Pancreatic Oncology, Tianjin Cancer Hospital Airport Hospital, National Clinical Research Center for Cancer, Tianjin 300308, China

† These authors contributed equally.

* To whom correspondence may be addressed.

Emails: yangxiaoying@tmu.edu.cn (Xiaoying Yang); wangyinsong@tmu.edu.cn (Yinsong Wang); yang_liu@tmu.edu.cn (Yang Liu).

**Experimental Methods**

*Cellular Internalization and Intracellular Drug Release of DOX@CWV in Cancer Cells:* Firstly, the cellular uptake of DOX@CWV was assessed in SCC-7 cells using flow cytometry. The cells were seeded into 12-well plates at a cell density of 1×10^5^ cells mL^‒1^ and cultured for 24 h. Then, these cells were incubated with DOX and DOX@CWV separately for 1, 2, 4, and 8 h at the same DOX concentrations of 1 μg mL^‒1^. Next, the treated cells were collected, washed with PBS, and analyzed using a flow cytometry (FACSVerse, BD, USA) for detecting the intracellular fluorescence of DOX. To investigate the entry mechanism of DOX@CWV, various endocytosis inhibitors—chlorpromazine (CPZ), methyl-β-cyclodextrin (M-β-CD), filipin III, and amiloride—were used to assess whether DOX@CWV entered SCC-7 cells via the endocytic pathway. Briefly, SCC-7 cells were seeded into 12-well plates at a density of 1×10⁵ cells/mL and cultured for 24 hours. The cells were then pretreated with the respective inhibitors for 30 minutes before being exposed to a fixed concentration of DOX@CWV for 4 hours. After incubation, the treated cells were collected, washed with PBS, and analyzed by flow cytometry.

Next, the release of DOX from DOX@CWV in SCC-7 cells was evaluated by observing the intracellular localization of DOX under a confocal microscopy. Briefly, the cells were incubated with DOX@CWV at the DOX concentration of 1 μg mL^‒1^ for 1, 2, and 4 h respectively, and then their nuclei were stained with 4',6-diamidino-2-phenylindole (DAPI, Solarbio, Beijing, China). After that, the intracellular localization of DOX in these cells were observed under a confocal microscope (Axio-Imager LSM-800, Zeiss, German). We also evaluated the influence of ultrasonic (US) irradiation (1 MHz, 1.5 W cm^‒2^) on the intracellular release of DOX. After incubation with DOX@CWV for 4 h, the cells were exposed to an ultrasound for 5 min and further incubated for 1, 2, and 4 h, respectively. These cells were stained with DAPI and finally observed under a confocal microscope.

*POD-Mimicking Activity of DOX@CWV in Cancer Cells:* The H_2_O_2_ decomposition activity of DOX@CWV was measured in SCC-7 cells by using [Ru(dpp)_3_]Cl_2_ as a luminescent oxygen sensor. Briefly, the cells were seeded into 12-well plates at a cell density of 1×10^5^ cells mL^‒1^ and cultured for 24 h. These cells were then incubated separately with DOX, CWV, DOX@CWV, and DOX@CWV+H_2_O_2_ for 4 h. Here, the final concentrations of DOX, CWV, and H_2_O_2_ were 1 μg mL^‒1^, 5×10^5^ Pg cells mL^‒1^, and 100 μmol L^‒1^ separately. The treated cells were processed with [Ru(dpp)_3_]Cl_2_ according to the manufacture’s instruction and next stained with DAPI. The luminescence of [Ru(dpp)_3_]Cl_2_ in these cells were observed under a confocal microscopy.

The GSH consumption ability of DOX@CWV was evaluated by detecting the intracellular GSH content using Ellman's reagent. Briefly, the cells were seeded into 12-well plates at a cell density of 1×10^5^ cells mL^‒1^ and cultured for 24 h. These cells were incubated separately with free DOX, CWV, and DOX@CWV for 24 h at the DOX and CWV concentrations of 1 μg mL^‒1^ and 5×10^5^ Pg cells mL^‒1^, and then lysed with the cell lysis buffer. The lysates thus obtained were processed with 0.2 mmol L^‒1^ DTNB and their absorbance values at 412 nm were measured with an UV-Vis spectrophotometer. Subsequently, the GSH contents were calculated according to the manufacture’s protocol.

*Sonodynamic Activity of DOX@CWV in Cancer Cells:* 2’,7’-Dichlorofluorescein diacetate (DCFH-DA) was used as a fluorescence probe to evaluate the intracellular generation of ROS in SCC-7 cells. Briefly, the cells were seeded into 12-well plates at a cell density of 1×10^5^ cells mL^‒1^ and cultured for 24 h. These cells were then incubated separately with DOX, CWV, DOX@CWV, and DOX@CWV+H_2_O_2_ for 4 h. Here, the final concentrations of DOX, CWV, and H_2_O_2_ were 0.5 μg mL^‒1^, 2.5×10^5^ Pg cells mL^‒1^, and 100 μmol L^‒1^, respectively. Next, the cells in the US irradiation groups were exposed to ultrasound (1 MHz, 1.5 W cm^‒2^) for 5 min. All the cells with and without US irradiation were incubated for another 12 h and then processed with DCFH-DA (Sigma-Aldrich, St Louis, USA). The intracellular DCF produced from DCFH-DA were quantitatively analyzed with a flow cytometer, and also observed under a confocal microscope after staining with DAPI.

Rhodamine 123 (Rh123) was used as a fluorescence indicator to evaluate the ROS-induced damage of mitochondria in SCC-7 cells. Briefly, the cells were seeded into confocal dishes at a density of 1×10^5^ cells mL^‒1^ and given the treatments as mentioned above. The treated cells were processed with Rh 123 (Sigma-Aldrich, St Louis, USA) and Hoechst 33342 (Beyotime Biotechnology, Shanghai, China) according to the manufactures’ instructions, and then observed the fluorescence signals of Rh123 under a confocal microscopy.

*Synergistic Anticancer Effects of DOX@CWV in Cancer Cells:* Firstly, MTT assay was used to evaluate the biosafety and the influence of US irradiation (1 MHz) on the cell growth at different power densities and duration times. The NIH-3T3 cells were seeded in a 96-well plate (5×10^3^ cells per well) and cultured for 24 h. Then, CWV at the concentrations of 0.5, 1.25, 2.5, 3.75 and 5×10^5^ Pg cells mL^‒1^ was respectively added to cells and incubated for another 24 h. The SCC-7 cells were seeded into 96-well plates at a cell density of 5×10^3^ cells mL^‒1^ and cultured for 24 h. Then, these cells were processed with US irradiation for 5 min at the power densities of 0.5, 1.0, 1.5, 2.0, and 2.5 W cm^‒2^, as well as for 1, 2, 3, 4, and 5 min at the power density of 1.5 W cm^‒2^. All the cells were cultured for another 24 h and then processed with MTT reagent (Meilun Biology Technology, Dalian, China). The formazan produced in these cells were dissolved in DMSO, and their absorbance at 490 nm were detected using a full wavelength microplate reader (Multiskan GO, Thermo Fisher Scientific, USA) for calculating the cell viabilities.

MTT assay was also used to evaluate the synergistic cytotoxicity of DOX@CWV-mediated combination treatment in SCC-7 cells. The cells were inoculated in 96-well plates at a cell density of 5×10^3^ cells mL^‒1^ and cultured for 24 h. Then, these cells were incubated separately with different concentrations of DOX, CWV, DOX@CW, and DOX@CWV+H_2_O_2_ for 4 h. Here, the concentrations of DOX were 0.125, 0.25, 0.5, 0.75, and 1 μg mL^‒1^, the concentrations of CWV were 0.5, 1.25, 2.5, 3.75, and 5×10^5^ Pg cells mL^‒1^, and the concentration of H_2_O_2_ was 100 μmol L^‒1^. The cells in the US irradiation groups were next exposed to ultrasound (1 MHz, 1.5 W cm^‒2^) for 5 min and processed with MTT reagent to detect the cell viabilities. In the meantime, the cells receiving the above treatments were stained with Live/Dead cell staining kit (Meilun Biology Technology, Dalian, China) and then observed under a fluorescence microscope (Axio Observer3, ZEISS, German). Hence, the cytotoxicity of the above treatments could be visualized by observing the red and green fluorescence.

Synergistic apoptosis-inducing effect was further evaluated in SCC-7 cells. The cells were seeded into 12-well plates at a density of 1×10^5^ cells mL^‒1^ and cultured for 24 h. These cells were then incubated separately with free DOX, CWV, DOX@CWV, and DOX@CWV+H_2_O_2_ for 4 h at the DOX, CWV, and H_2_O_2_ concentrations of 0.5 μg mL^‒1^, 2.5×10^5^ Pg cells mL^‒1^, and 100 μmol L^‒1^, respectively. The cells in the US irradiation groups were exposed to ultrasound (1 MHz, 1.5 W cm^‒2^) for 5 min and then processed with an Annexin V-FITC/7-AAD apoptosis detection kit (Meilun Biology Technology, Dalian, China) according to the manufacture’s protocol. Subsequently, the apoptosic cells were analyzed using a flow cytometry.

We also evaluated the immunogenic cell death (ICD)-inducing effect in SCC-7 cells. The cells were seeded into 12-well plates at a density of 1.0×10^5^ cells mL^‒1^ and cultured for 24 h. These cells were given the same treatments as described above and incubated for another 24 h. For evaluating the surface exposure of calreticulin (CRT), the cells were processed with Alexa Fluor® 488 anti-CRT antibody (Bioss, Beijing, China) according to the manufacture’s protocol. The stained cells were further analyzed with a flow cytometry and observed under a confocal microscope. For evaluating the release of high-mobility group box-1 (HMGB1) from the cell nucleus into the cytoplasm, the treated cells were processed with rabbit anti-HMGB1 primary antibody (Bioss, Beijing, China) and FITC-labeled goat anti-rabbit secondary antibody (Thermo Fisher Scientific, Waltham, MA, USA) successively, and finally observed under a confocal microscopy. For evaluating the release of adenosine triphosphate (ATP), the treated cells were processed with an ATP assay kit (Beyotime Biotechnology, Shanghai, China) according to the manufacturer’s protocol and then analyzed with a Multifunctional Enzyme Labeler (Ensight, Revvity, Shanghai, China).

*Synergistic* *Immunostimulatory Effects in Immune Cells:* SCC-7 cells were given the above treatments including PBS (the control), DOX, CWV, and DOX@CWV, and their combination with US irradiation (1 MHz, 1.5 W cm^‒2^, 5 min). After incubation for 24 h, the culture media were harvested from these treated cells and centrifuged at 300 g for 10 min. The supernatants were collected and used as the tumoral antigens for the following experiments.

BMDCs, isolated from the bone marrow of healthy female C57BL/6 mice using a previous method,^[S1]^ were seeded into 6-well plates at a density of 5.0×10^5^ cells mL^‒1^ and then incubated with the tumoral antigens collected above for 48 h. Afterwards, these BMDCs were collected processed with the immunofluorescence staining of CD11c/CD83, CD11c/CD80/CD86, and CD11c/MHC-I/MHC-II by using anti-CD11c-PE, anti-CD83-FITC, anti-CD80-FITC, anti-CD86-APC, anti-MHC-I-APC, and anti-MHC-II-FITC antibodies (BioLegend, San Diego, CA, USA) separately according to the manufactures’ instructions, and subsequently analyzed with a flow cytometer.

T cells were isolated from the spleens of healthy female C57BL/6 mice using the method reported previously.^[S2]^ BMDCs and T cells were co-cultured in 12-well plates at an amount ratio of 1:5, and then incubated with the above tumoral antigens for 48 h. Then, T cells were collected and processed with immunofluorescent staining as follows. For evaluating the expression levels of CD69 (an early activation marker), T cells were stained with anti-CD3-FITC/anti-CD69-PE antibodies (BioLegend, San Diego, CA, USA). For evaluating the proportions of cytotoxic CD8^+^ T cells, T cells were stained with anti-CD3-FITC/anti-CD8-APC antibodies. These stained T cells were finally analyzed using a flow cytometer. Meanwhile, the supernatants of the above co-culture systems were collected, and the secretion levels of TNF-α, IFN-γ, and IL-12p70 were detected using the corresponding Elisa assay kits (ABclonal, Wuhan, China) in accordance with the manufactures’ instructions. Additionally, the Cell Counting Kit-8 (CCK-8) assay was used to assess the cytotoxicity of CWV on immune cells. T cells were seeded into a 96-well plate at a density of 1×10⁴ cells per well and cultured for 24 hours. CWV was then added at concentrations of 0.5, 1.25, 2.5, 3.75, and 5×10⁵ Pg/mL, followed by a 48-hour incubation. Afterward, the cells were treated with the CCK-8 reagent (Meilun Biology Technology, Dalian, China) according to the manufacturer’s protocol. The absorbance at 450 nm was measured using a full-wavelength microplate reader to determine cell viability.

A co-culture system of BMDCs, T cells, and SCC-7 cells was constructed using Transwell chamber with 0.4 μm pore polycarbonate membrane for evaluating the activation of specific anticancer immunity. BMDCs and splenic T cells were seeded into the upper chambers, and SCC-7 cells were seeded into the bottom chambers. The amount ratio of SCC-7 cells, BMDCs and T cells was 1:10:50. BMDCs and T cells in the upper chambers were incubated with the tumoral antigens collected above for 48 h. Afterwards, SCC-7 cells in the bottom chambers were collected and further processed with MTT reagent for detecting the viabilities.

Mouse macrophage RAW264.7 cells were seeded into 12-well plates at a density of 3.0×10^5^ cells mL^‒1^, and then incubated with the tumoral antigens collected above for 48 h. Next, RAW264.7 cells were collected after centrifuging at 300 g for 10 min and further stained with anti-CD80-FITC antibody (BioLegend, San Diego, CA, USA). Finally, the expression levels of CD80 (the biomarker of M1 macrophages) were analyzed with a flow cytometer, thus evaluated the M1 polarization of RAW264.7 cells.

*In Vivo Sonodynamic Performance:* DCFH-DA was used as a fluorescence probe of ROS to evaluate the sonodynamic performance in SCC-7 tumor-bearing mice. Briefly, the mice were divided randomly into 8 groups with 3 mice each group, including PBS (the control) ± US irradiation, DOX ± US irradiation, CWV ± US irradiation, and DOX@CWV ± US irradiation. These mice were administrated with 50 μL of sample solutions supplemented with DCFH-DA through intratumoral injection. The doses of DOX and CWV were 0.15 mg kg^‒1^ and 7.5×10^7^ Pg cells kg^‒1^, respectively. After 4 h, the mice in the US irradiation groups were exposed to ultrasound with a power density of 1.5 W cm^‒2^ at the tumor site for 5 min. At 8 h after US irradiation, the mice were euthanized and their tumors were isolated for frozen-section. Tumor sections thus obtained were stained with DAPI and the fluorescence of intratumoral DCF were observed under a fluorescence microscope.

*In Vivo Synergistic Anticancer Effects and Biosafety:* SCC-7 tumor-bearing mice were divided randomly into 8 groups with 5 mice each group and given the above treatments twice respectively on the 0th and 4th. Within 14 d after the beginning of treatments, the tumor volumes of these mice were measured every 2 d according to the following formula. The body weights of these mice were also detected at the same time. After that, the mice were euthanized, and their tumors were collected, photographed, and further weighed for comparison.

Volume = length × width^2^ / 2

Major organs (heart, liver, spleen, kidney and lung) and blood samples were also collected from the above mice. Tumors and organs were fixed with 4% paraformaldehyde, embedded in paraffin, and sliced into 4-µm-thick sections for the following examinations. Tumor sections were stained with anti-Ki67 and anti-CD31 primary antibodies (Abcam, Cambridge, UK) separately, followed by processing with secondary antibodies according to the manufacture’s protocols. Furthermore, organ and tumor sections were processed with hematoxylin and eosin (H&E) staining. All the above sections were next observed under a microscope. Blood samples were subjected to blood routine and biochemical examinations.

*In Vivo Immune Activation and Antimetastatic Effects:* Bilateral SCC-7 tumor-bearing mice were divided randomly into 8 groups with 8 mice each group and given the same treatments as described above at the primary tumor site. At 2 d after the first treatments, 3 mice were chosen randomly from each group and euthanized. Tumors and spleens were isolated from these mice and further digested to obtain single-cell suspensions. For evaluating the maturation of DCs, the cells obtained from both tumors and spleens were processed separately with the immunofluorescence staining of CD11c/CD83, CD11c/CD80/CD86, and CD11c/MHC-I/MHC-II by using anti-CD11c-PE, anti-CD83-FITC, anti-CD80-FITC, anti-CD86-APC, anti-MHC-I-APC, and anti-MHC-II-FITC antibodies. For evaluating the activation of T cells, the cells obtained from both tumors and spleens were stained with anti-CD3-FITC/anti-CD8-APC antibodies. For evaluating the polarization of macrophages, the cells obtained from tumors were stained with anti-F4/80-PE/anti-CD80-FITC antibodies. For evaluating the inhibition of regulatory T cells, the cells obtained from spleens were stained with anti-CD4-PE/anti-CD25-FITC/anti-Foxp3-APC antibodies. Finally, all the cells with the above staining were analyzed using a flow cytometer.

Within 14 d after the beginning of treatments, the volumes of distant tumors were measured for evaluating the metastasis. Next, these mice were euthanized, and their distant tumors and spleens were isolated for the following examinations. Distant tumors were frozen-sectioned for evaluating the activation of T cells. Frozen sections were stained successively with anti-CD8 primary antibody (Bioss, Beijing, China) and FITC-labeled goat anti-rabbit secondary antibody, and then observed under a fluorescence microscope. Spleens were digested to obtain single-cell suspensions for evaluating the immune memory response. The cells thus obtained were stained with anti-CD3-FITC/anti-CD44-PE/anti-CD62L-APC antibodies and then analyzed with a flow cytometry.

**Reference**

[S1] Y. Cheng, Q. Chen, Z. Guo, M. Li, X. Yang, G. Wan, H. Chen, Q. Zhang, Y. Wang, *ACS Nano.* **2020**, *14*, 15161‒15181.

[S2] Y. Cheng, Q. Chen, Z. Qian, T. Shan, L. Bai, X. Jiang, C. Li, Y. Wang, *Adv. Healthc. Mater*. **2023**, *12*, e2201690.

**Table S1.** Encapsulation efficiencies of DOX in CWV derived from 1.5×10^7^ Pg cells at different input amounts of DOX

| Input amounts of DOX  (μg) | Encapsulated amounts of DOX  (μg) | Encapsulation efficiency (%) |
| --- | --- | --- |
| 7.5 | 6.9±0.3 | 92.4±3.8 |
| 15 | 13.0±0.3 | 86.5±1.8 |
| 30 | 25.6±0.6 | 85.4±2.1 |
| 75 | 61.5±0.9 | 82.0±1.2 |
| 150 | 91.5±4.5 | 61.0±3.0 |

**Figures**

**
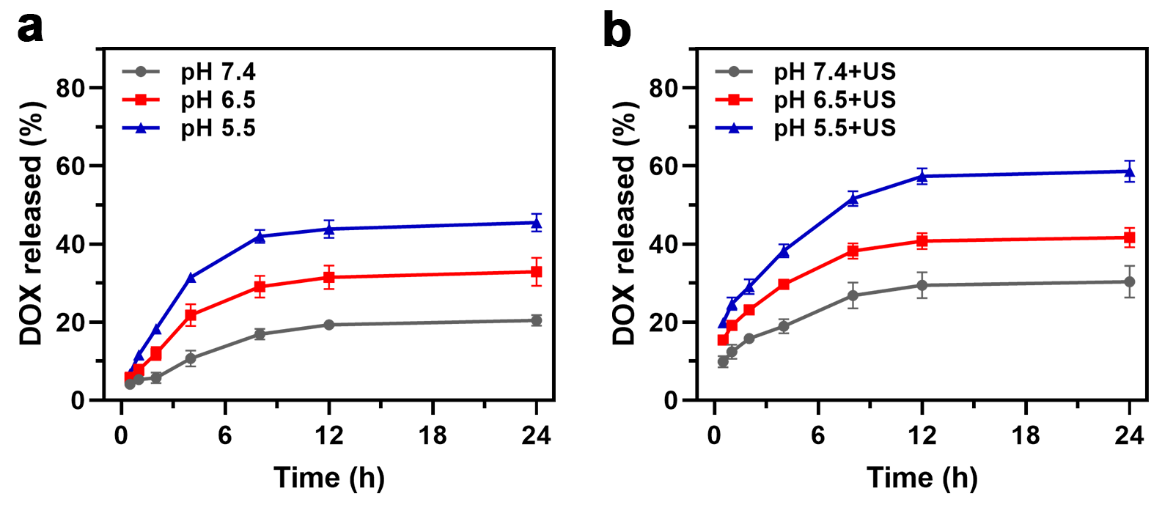
**

**Figure S1.** pH and US-responsive drug release properties of DOX@CWV. a) In vitro release profiles of DOX from DOX@CWV and b) DOX@CWV preprocessed with US irradiation at different pH values. Data are presented as mean values ± SD (n=3). US irradiation (1 MHz, 1.5 W cm^‒2^) was performed for 5 min.


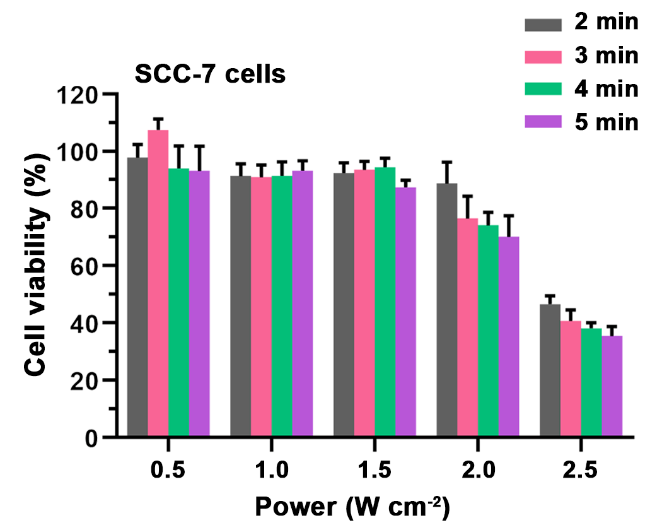


**Figure S2.** Effect of different power densities of ultrasonic irradiation on the growth of SCC-7 cells determined using MTT assay. Data are presented as mean ± SD (n = 3).


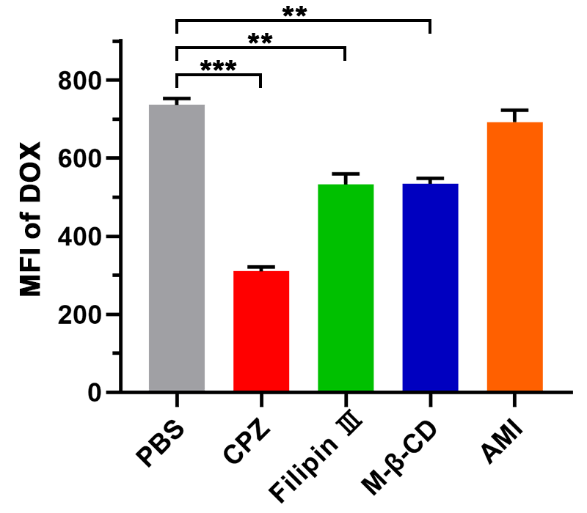


**Figure S3.** Inhibition of DOX@CWV intracellular uptake in SCC-7 cells by various endocytosis inhibitors. Data are presented as means ± SD (n = 3). ** and *** represent p < 0.01 and < 0.001 between two treatment groups (One-way ANOVA).

**
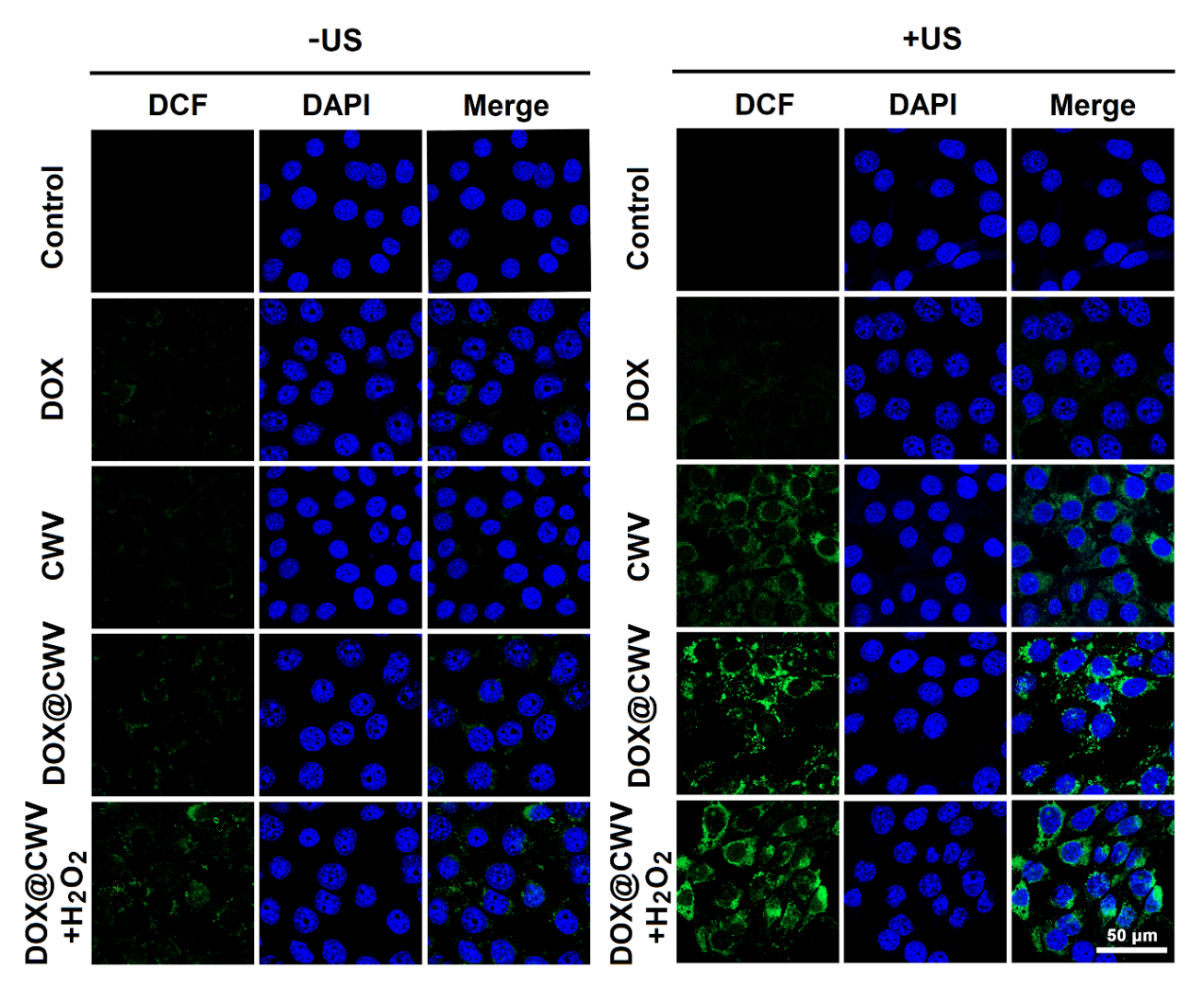
**

**Figure S4.** Confocal images of SCC-7 cells processed with DCFH-DA after the treatments of DOX ± US, CWV ± US, DOX@CWV ± US, and DOX@CWV+H_2_O_2_ ± US. The concentrations of DOX and CWV were 0.5 μg mL^‒1^ and 2.5×10^5^ Pg cells mL^‒1^, respectively. US irradiation (1 MHz, 1.5 W cm^‒2^) was performed for 5 min. Scale bar: 50 μm.


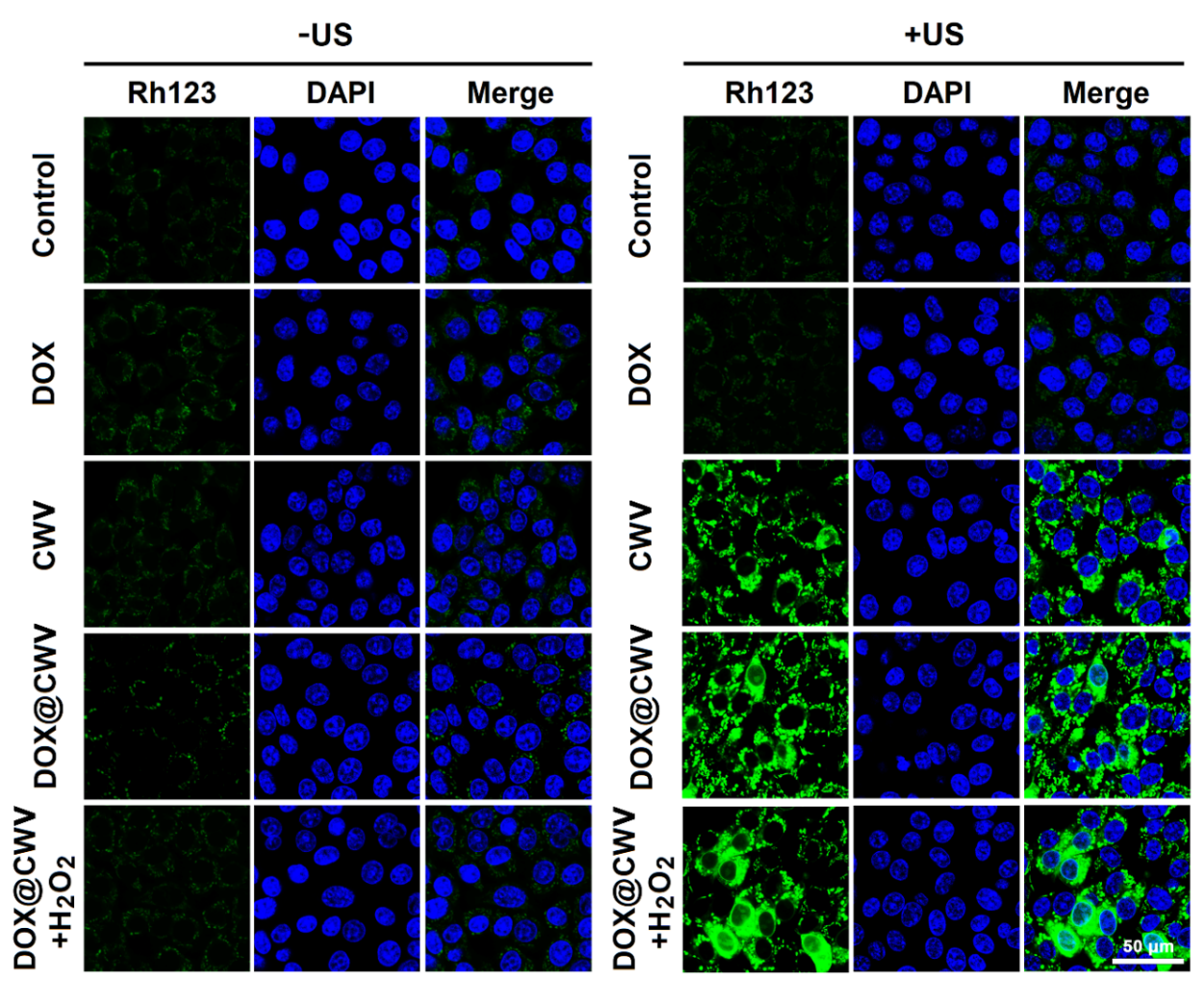


**Figure S5.** Confocal images of SCC-7 cells processed with Rh123 after the treatments of DOX ± US, CWV ± US, DOX@CWV ± US, and DOX@CWV+H_2_O_2_ ± US. US irradiation (1 MHz, 1.5 W cm^‒2^) was performed for 5 min. Scale bar: 50 μm.


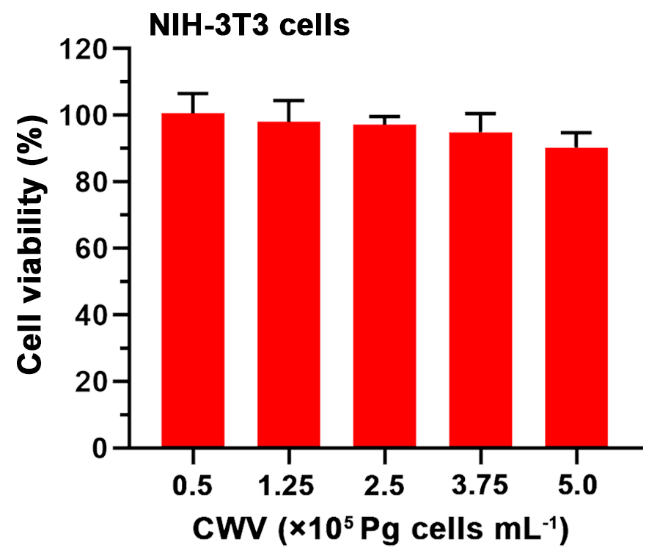


**Figure S6.** Biosafety of CWV at different concentrations in NIH-3T3 cells determined using MTT assay. Data are presented as mean ± SD (n = 3).


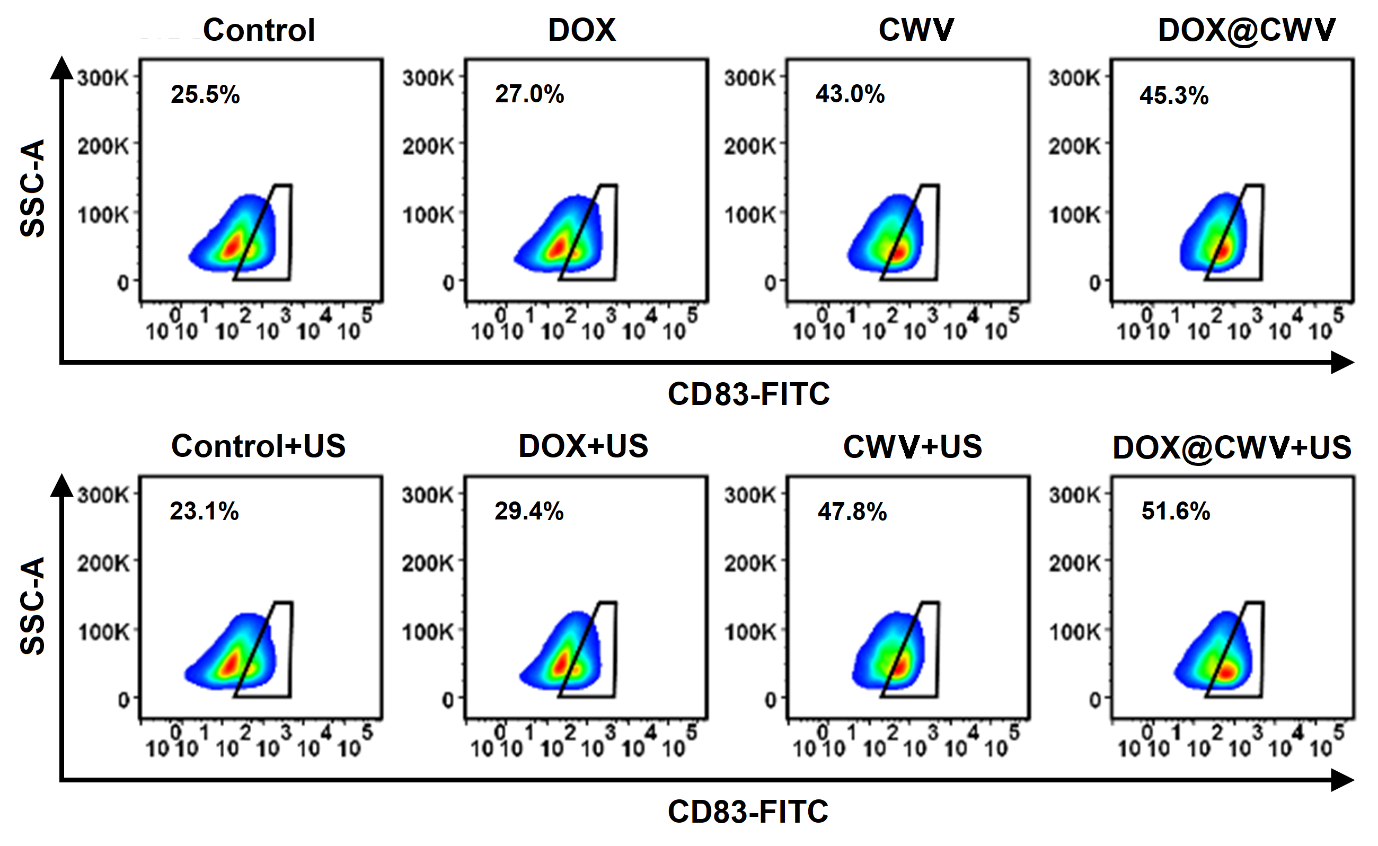


**Figure S7.** Flow cytometric plots of CD83^+^ CD11c^+^ BMDCs after 48-hour incubation with the tumoral antigens obtained from SCC-7 cells receiving the treatments of DOX ± US, CWV ± US, DOX@CWV ± US, and DOX@CWV+H_2_O_2_ ± US. US irradiation (1 MHz, 1.5 W cm^‒2^) was performed for 5 min.


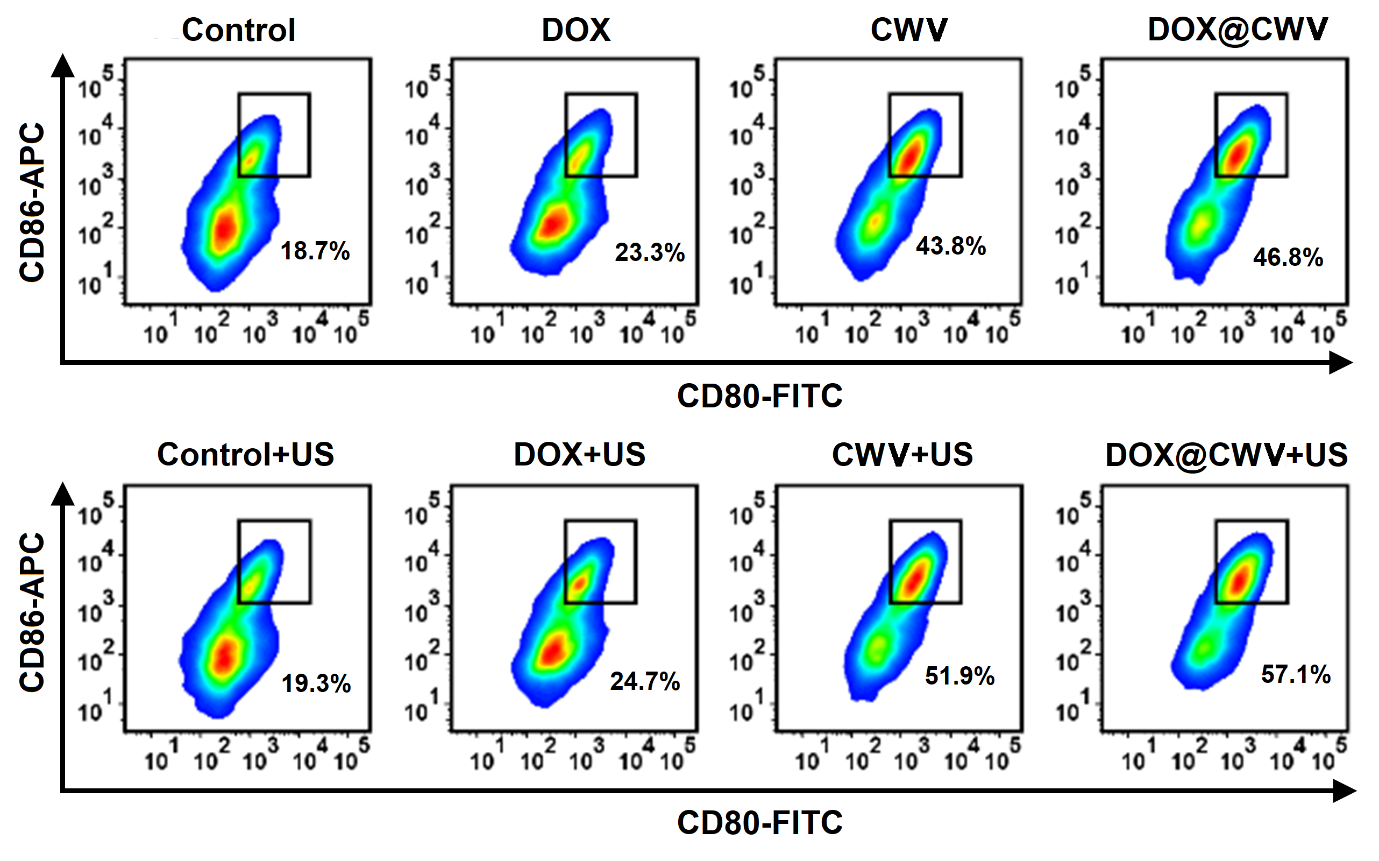


**Figure S8.** Flow cytometric plots of CD80^+^ CD86^+^ CD11c^+^ BMDCs after 48-hour incubation with the tumoral antigens obtained from SCC-7 cells after various treatments.


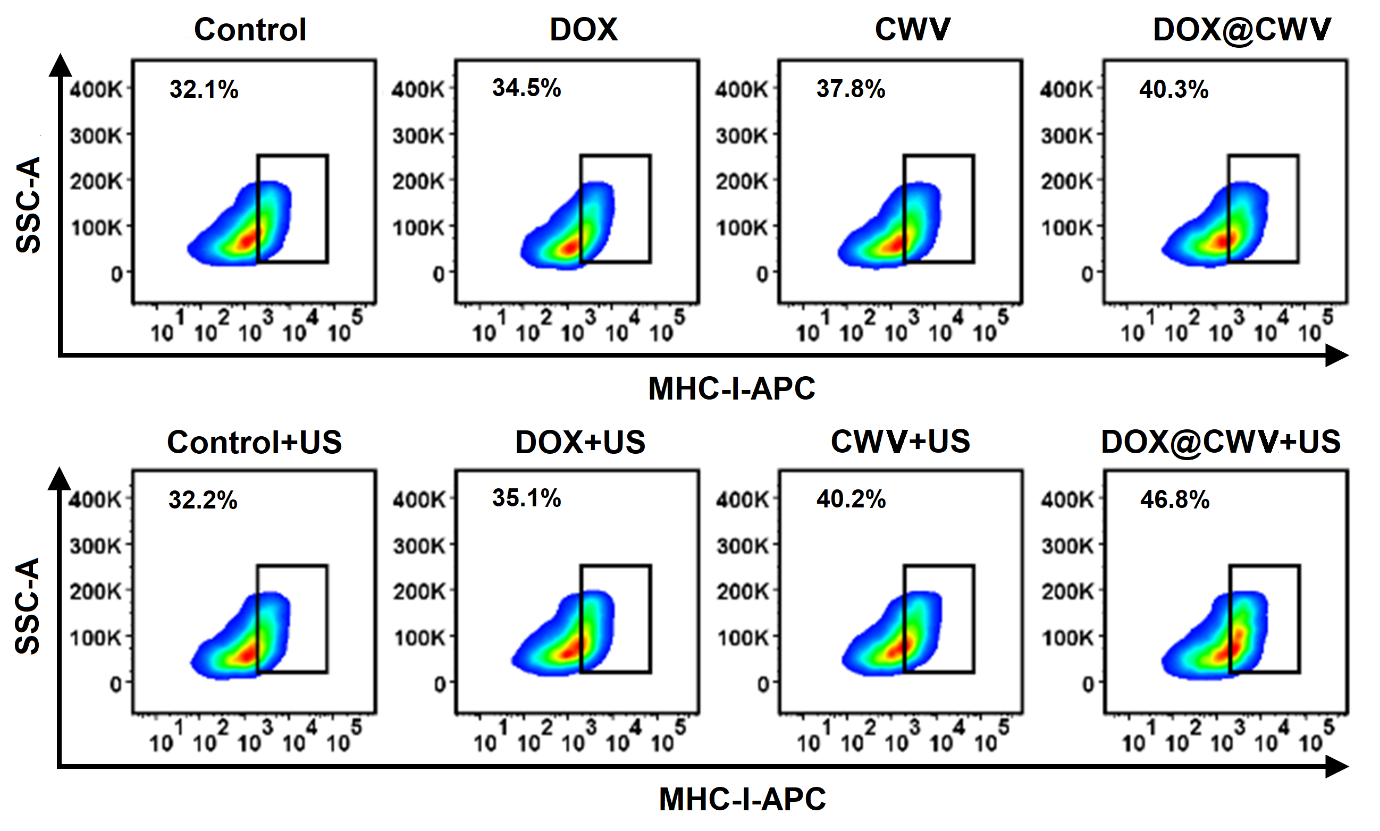


**Figure S9.** Flow cytometric plots of MHC-I^+^ CD11c^+^ BMDCs after 48-hour incubation with the tumoral antigens obtained from SCC-7 cells after various treatments.


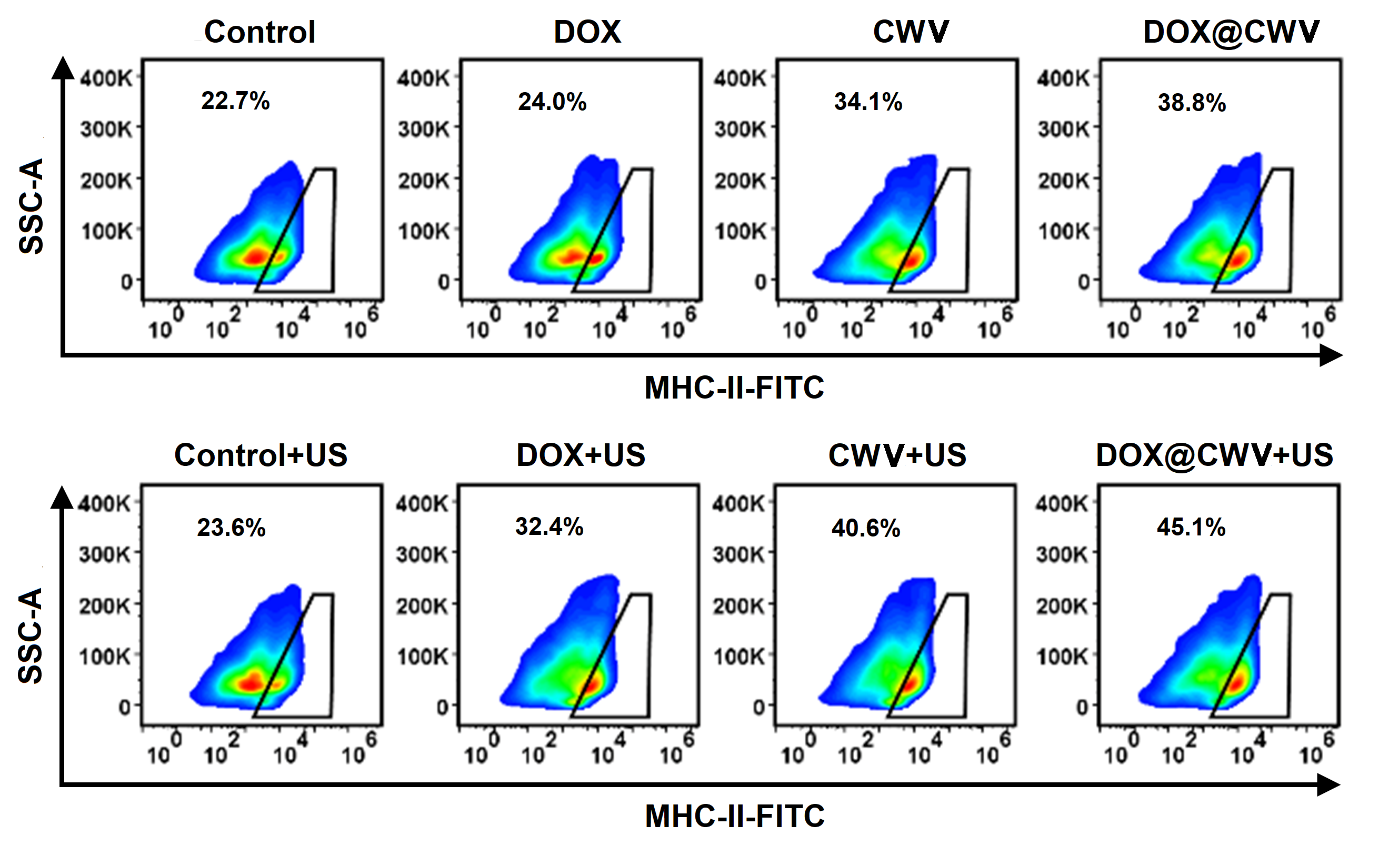


**Figure S10.** Flow cytometric plots of MHC-II^+^ CD11c^+^ BMDCs after 48-hour incubation with the tumoral antigens obtained from SCC-7 cells after various treatments.


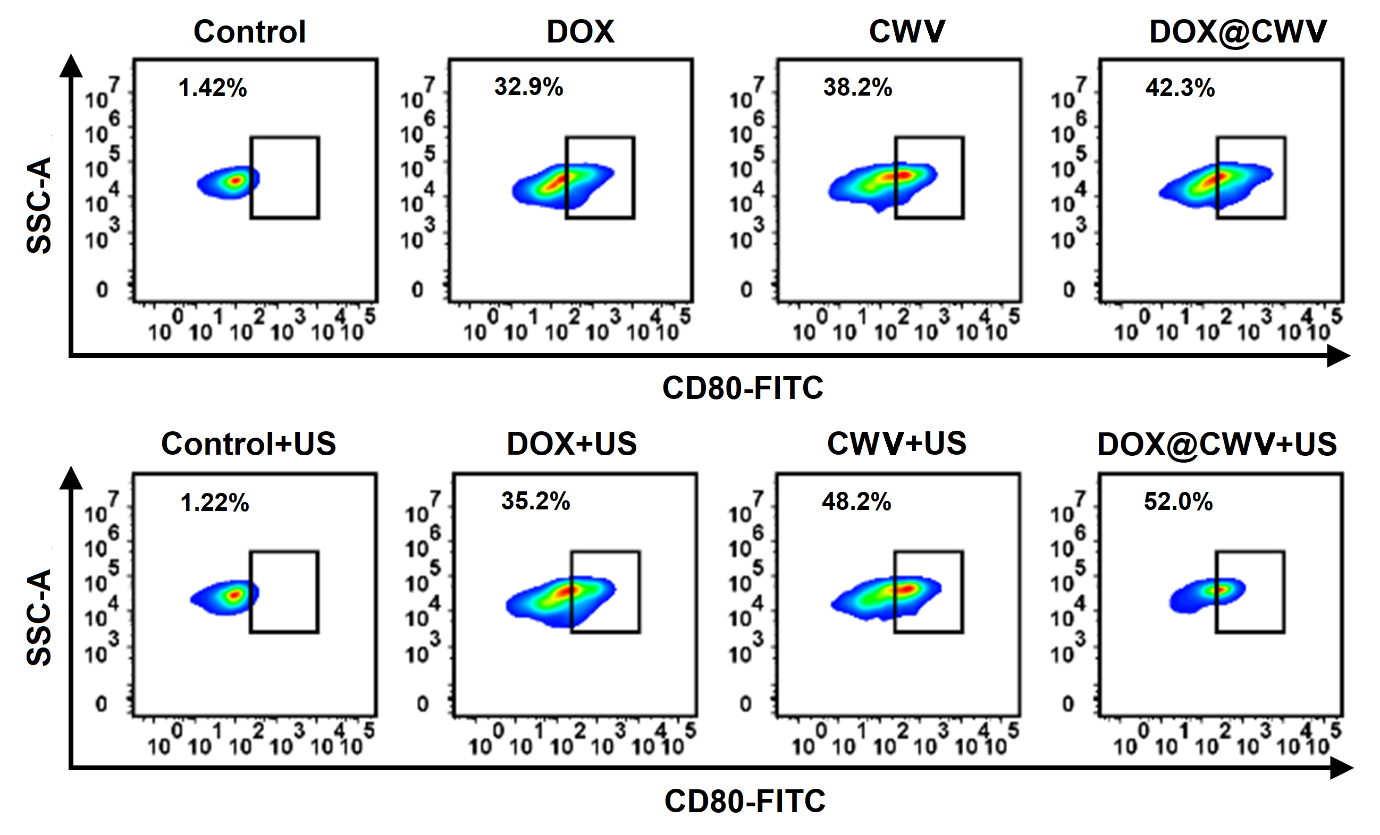


**Figure S11.** Flow cytometric plots of CD80^+^ RAW264.7 cells after 48-hour incubation with the tumoral antigens obtained from SCC-7 cells after various treatments.


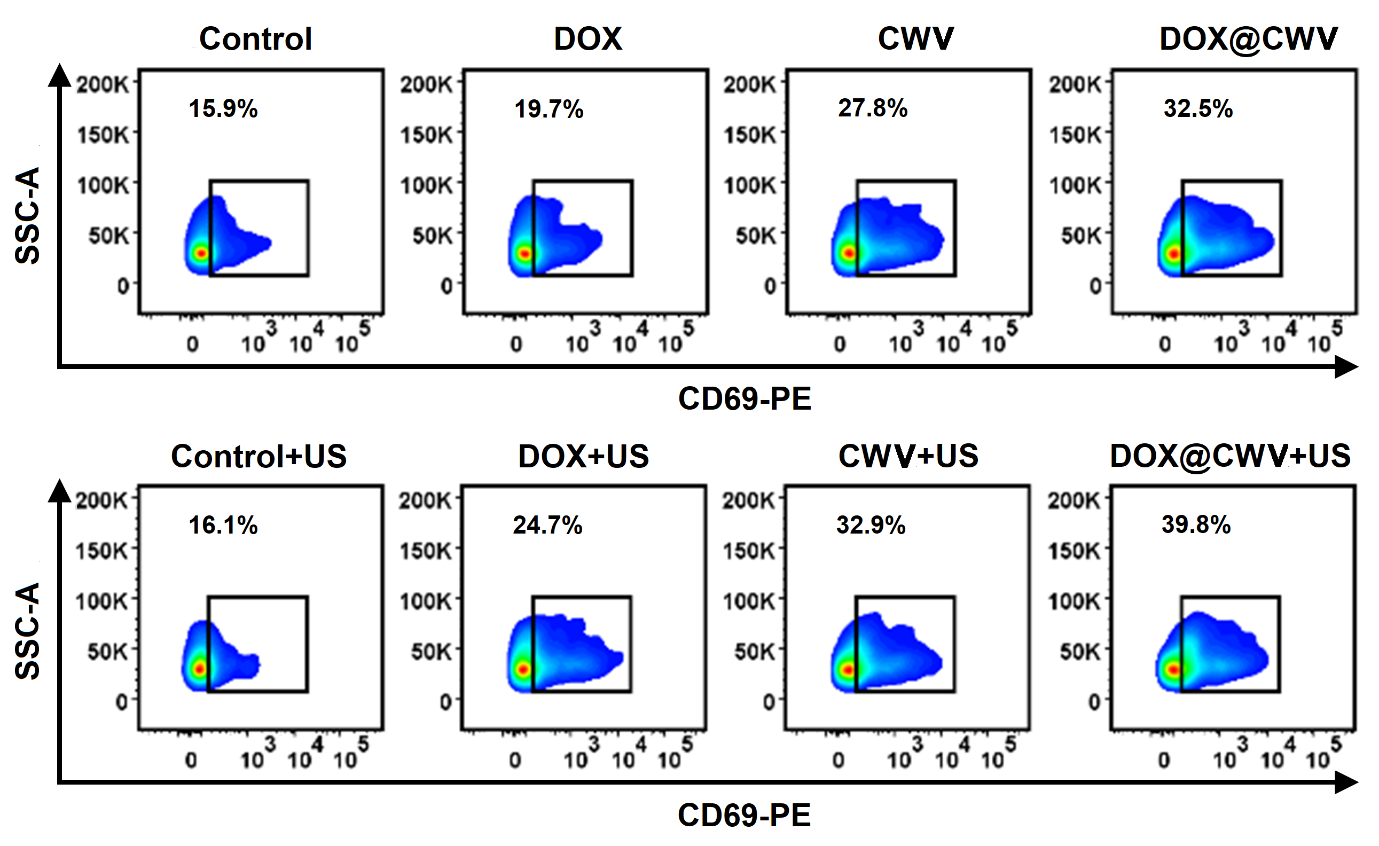


**Figure S12.** Flow cytometric plots of CD69^+^ CD3^+^ splenic T cells after 48-hour incubation with BMDCs and the tumoral antigens obtained from SCC-7 cells after various treatments.


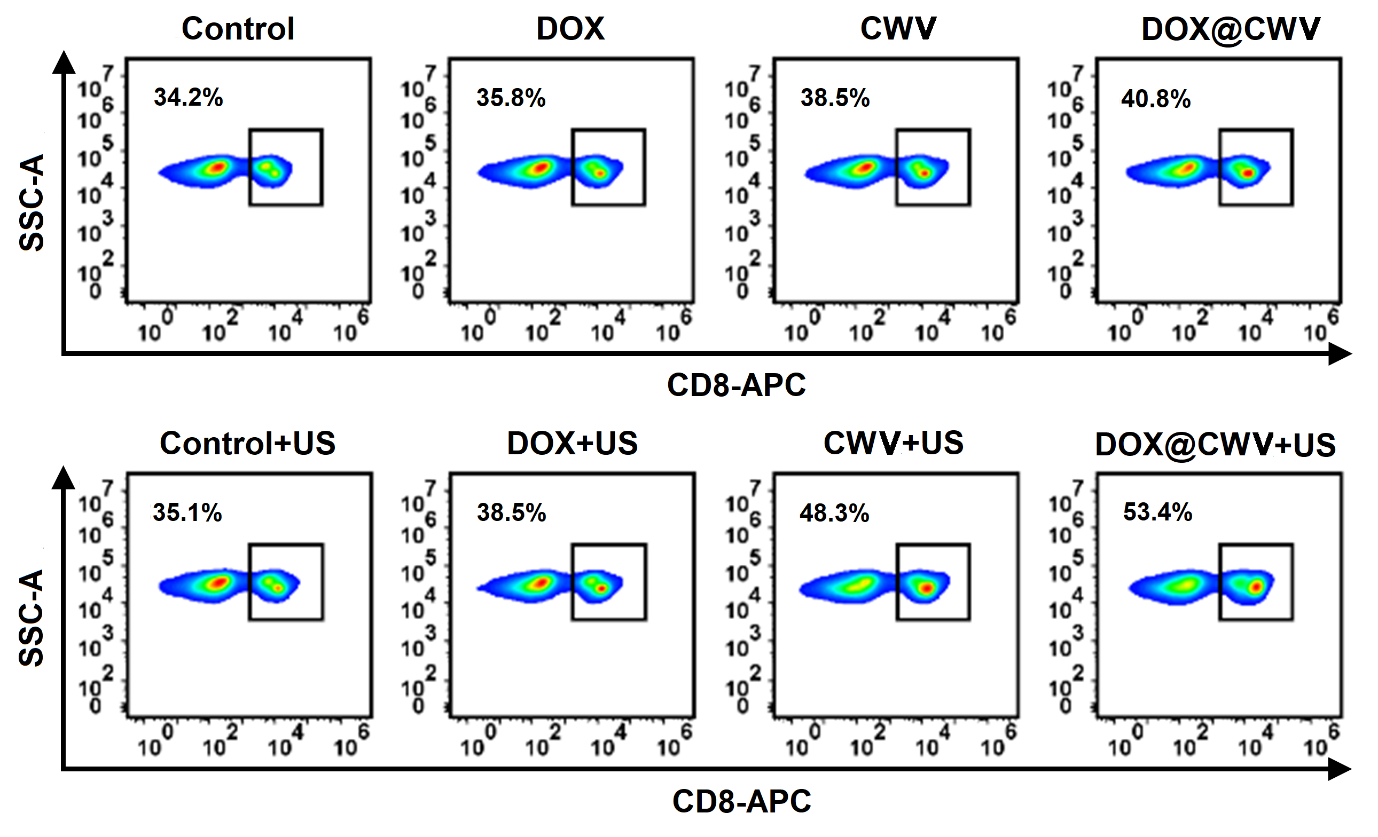


**Figure S13.** Flow cytometric plots of CD8^+^ CD3^+^ splenic T cells after 48-hour incubation with BMDCs and the tumoral antigens obtained from SCC-7 cells after various treatment


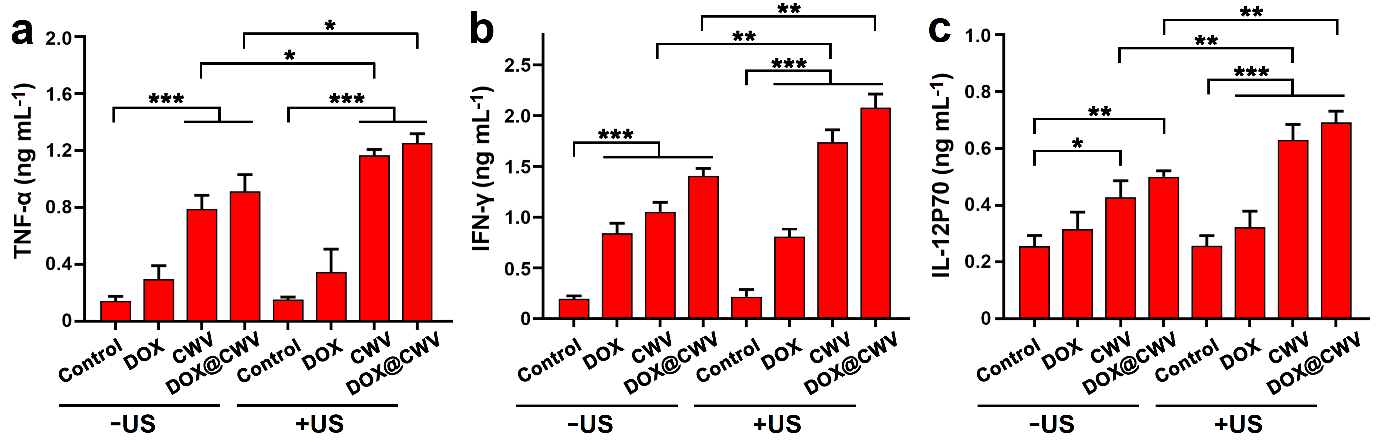


**Figure S14.** Secretion levels of a) TNF-𝛼, b) IFN-𝛾, and c) IL-12p70 determined with ELISA kits in the co-culture systems of BMDCs and Splenic T cells after 48-hour incubation with the tumoral antigens obtained from SCC-7 cells after various treatments. Data are presented as mean values ± SD (n = 3). *, **, and *** represent p < 0.05, < 0.01, and < 0.001 between two treatment groups (One-way ANOVA).


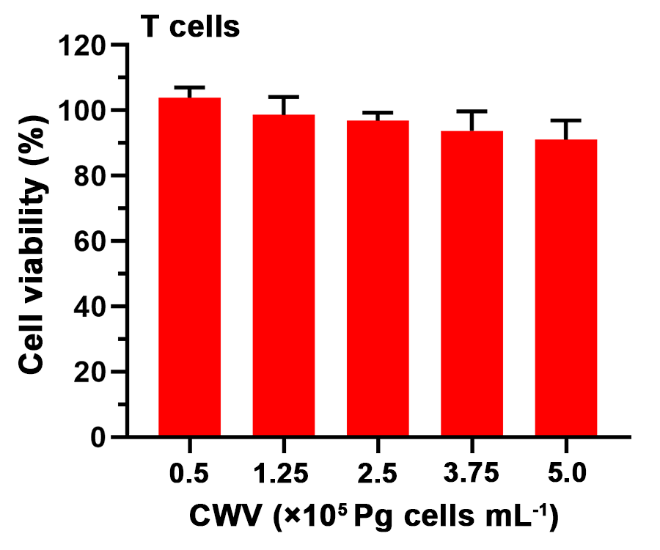


**Figure S15.** Cytotoxicity of CWV at varying concentrations in T cells, as determined by the CCK-8 assay. Data are presented as mean ± SD (n = 3).


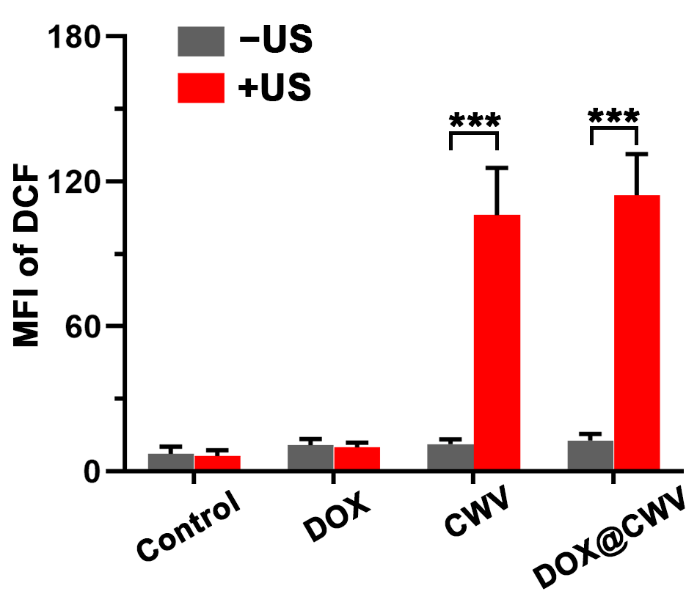


**Figure S16.** Comparison of the mean fluorescent intensity (MFI) of DCF in tumor sections. Data are shown as mean values ± SD (n = 3). *** represent p < 0.001 between two treatment groups (One-way ANOVA).


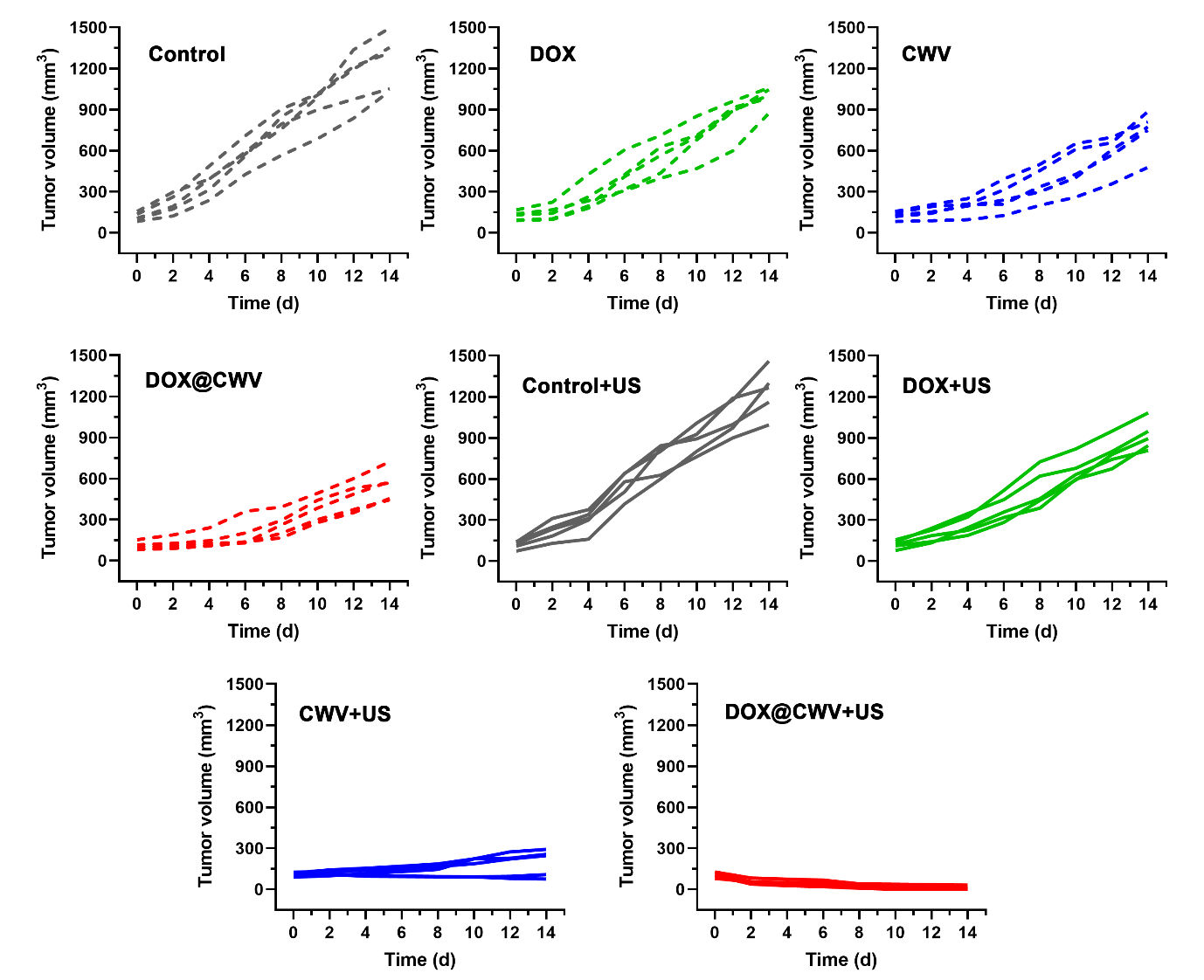


**Figure S17.** Individual tumor growth curves of SCC-7 tumor-bearing mouse within 14 d after the treatments of PBS ± US (the control ± US), DOX ± US, CWV ± US, and DOX@CWV ± US. The mice were administered with sample solutions through intratumoral injection. The doses of DOX and CWV were 0.15 mg kg^‒1^ and 7.5×10^7^ Pg cells kg^‒1^, respectively. US irradiation (1 MHz, 1.5 W cm^‒2^) was performed at the tumor site for 5 min.


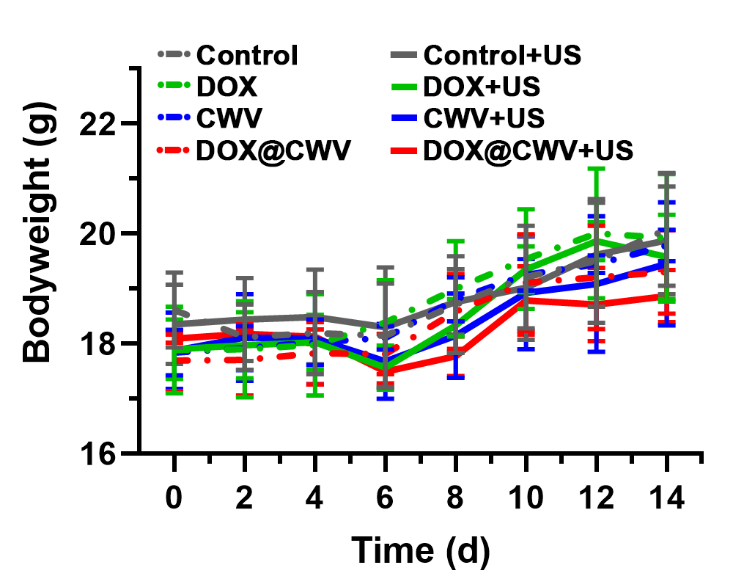


**Figure S18.** Body weights of SCC-7 tumor-bearing mice within 14 d after various treatments.


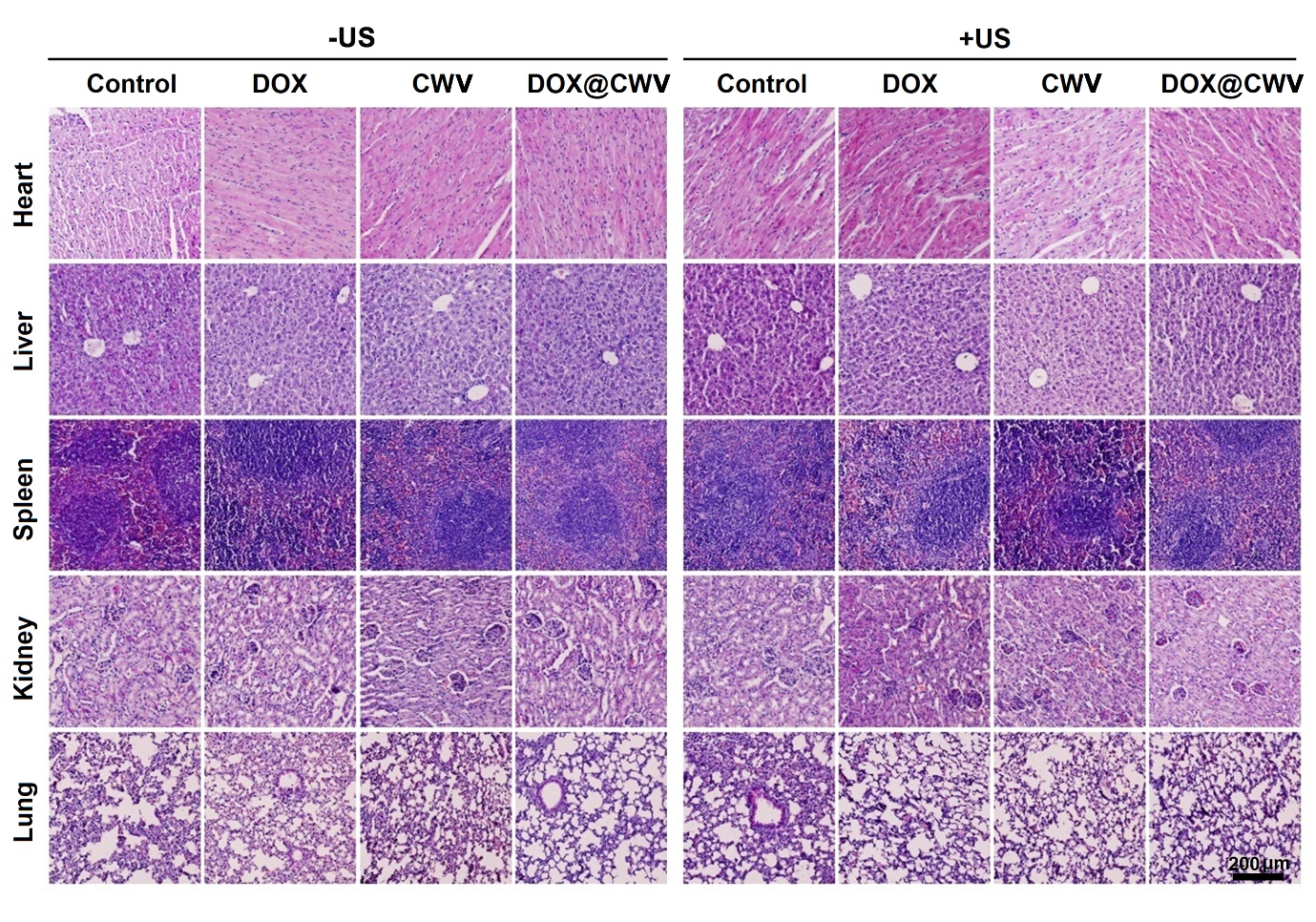


**Figure S19.** Microscope images of H&E-stained sections of major organs of SCC-7 tumor-bearing mice after various treatments. Scale bar: 200 μm.


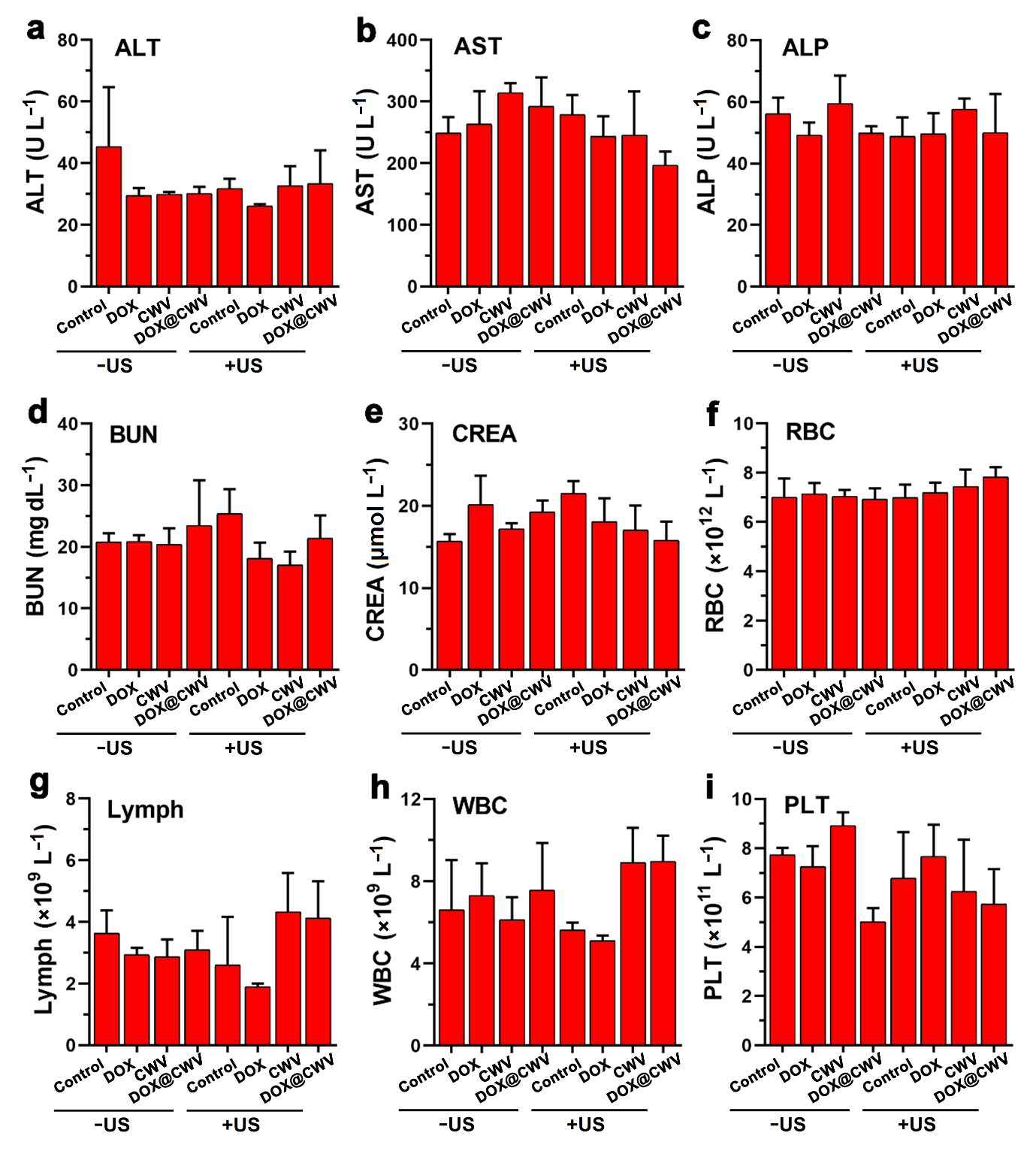


**Figure S20.** Results of routine blood examination and blood biochemistry analysis including a) alkaline alanine aminotransferase (ALT), b) aspartate aminotransferase (AST), c) phosphatase (ALP), d) blood urea nitrogen (BUN), e) creatinine (CREA), f) red blood cells (RBC), g) lymph, h) white blood cells (WBC), and i) platelets (PLT) in SCC-7 tumor-bearing mice after various treatments. Data are shown as mean values ± SD (n = 3).


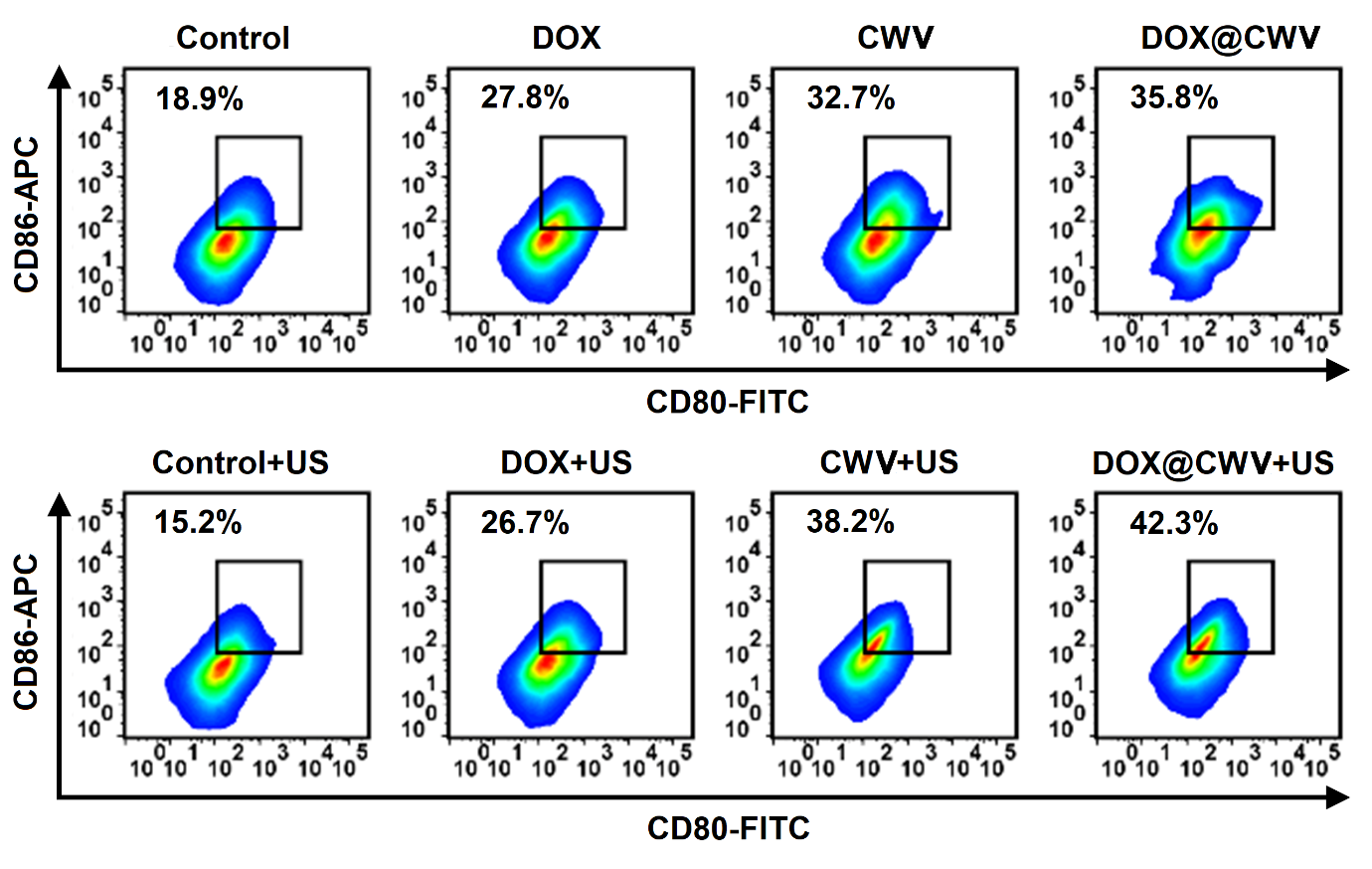


**Figure S21.** Flow cytometric plots of CD80^+^ CD86^+^ CD11c^+^ DCs in tumor tissues isolated from SCC-7 tumor-bearing mice after various treatments.


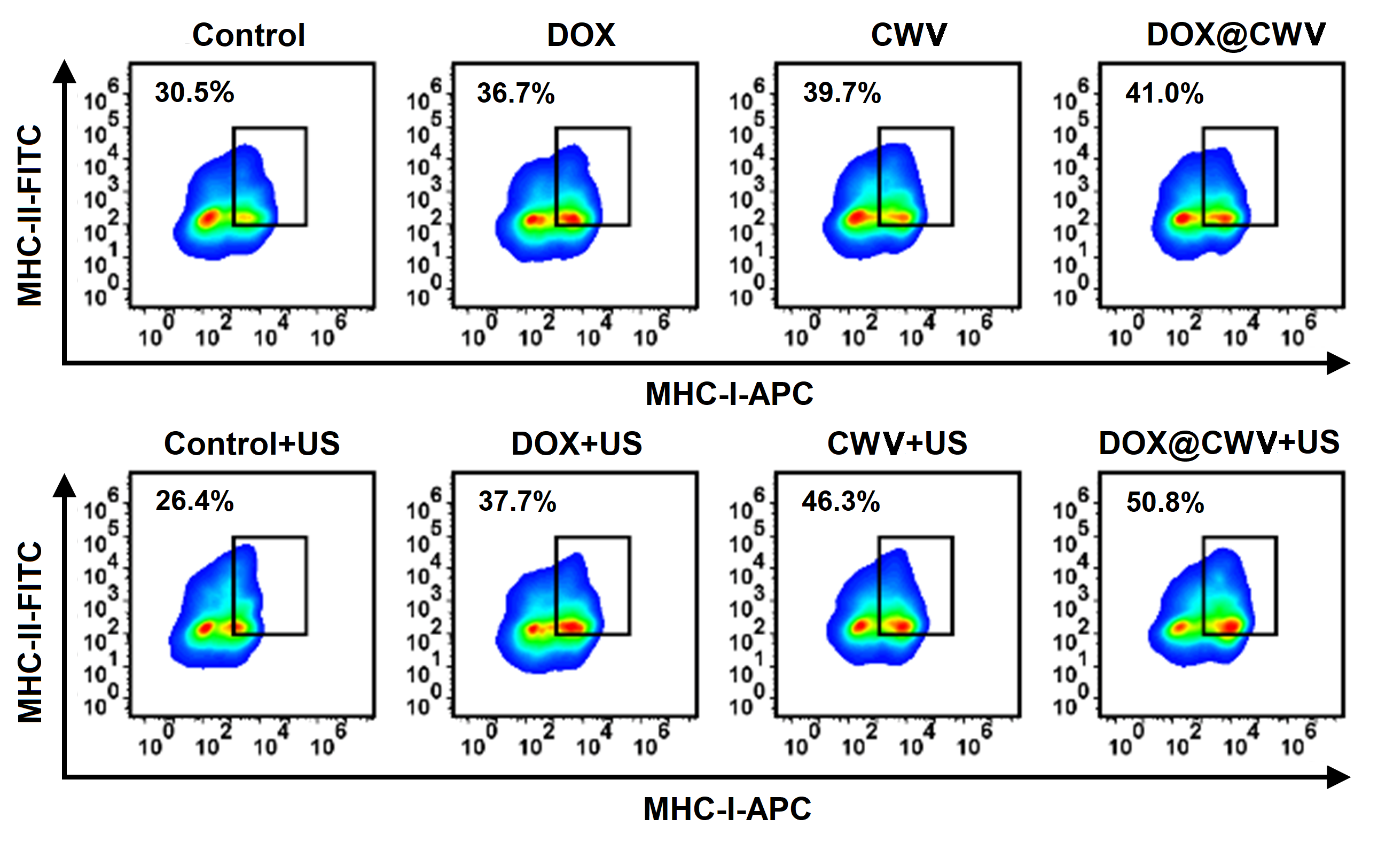


**Figure S22.** Flow cytometric plots of MHC-I^+^ MHC-II^+^ CD11c^+^ DCs in tumor tissues isolated from SCC-7 tumor-bearing mice after various treatments.


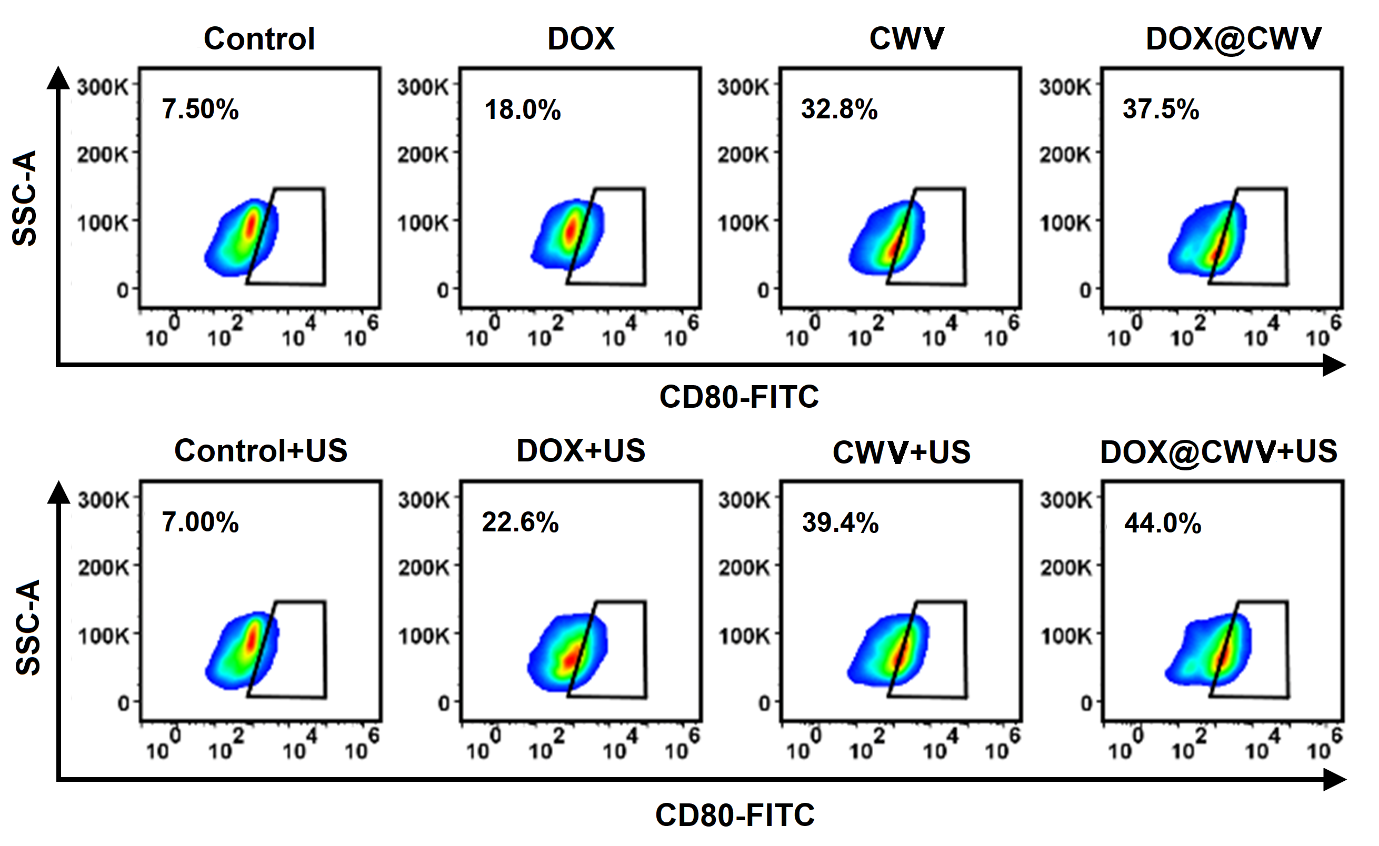


**Figure S23.** Flow cytometric plots of CD80^+^ F4/80^+^ macrophages in tumor tissues isolated from SCC-7 tumor-bearing mice after various treatments.


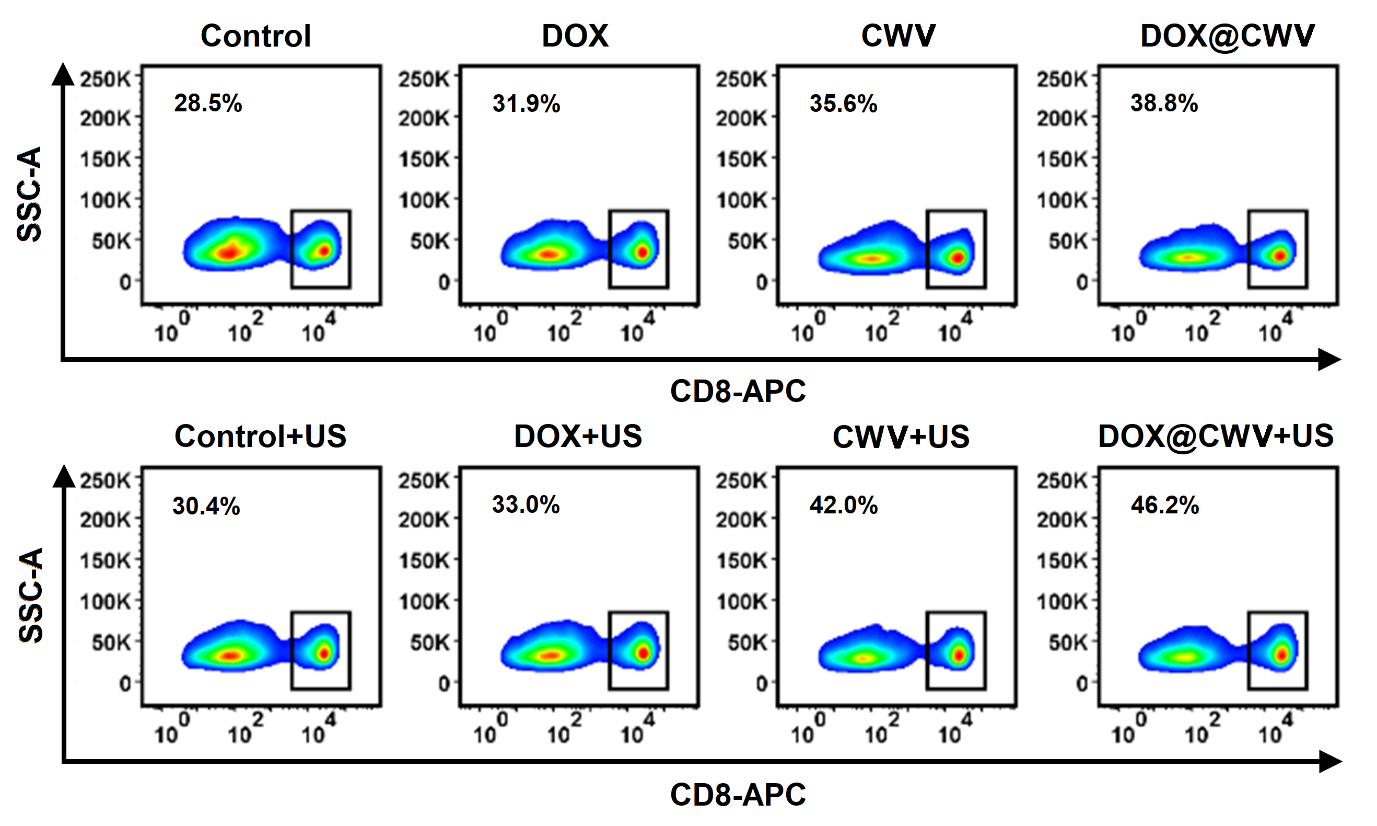


**Figure S24.** Flow cytometric plots of CD8^+^ CD3^+^ T cells in tumor tissues isolated from SCC-7 tumor-bearing mice after various treatments.


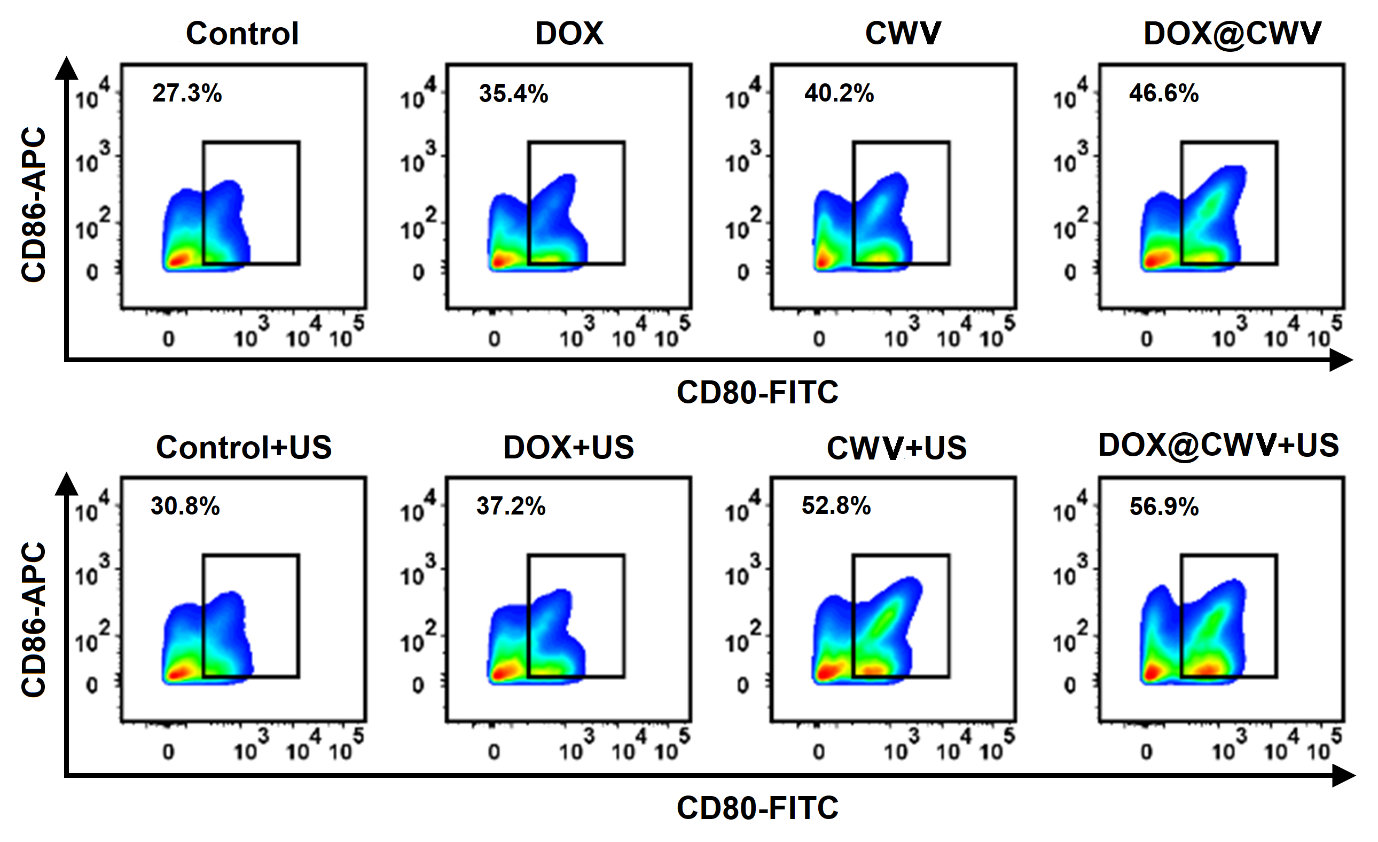


**Figure S25.** Flow cytometric plots of CD80^+^ CD86^+^ CD11c^+^ DCs in splenic tissues isolated from SCC-7 tumor-bearing mice after various treatments.


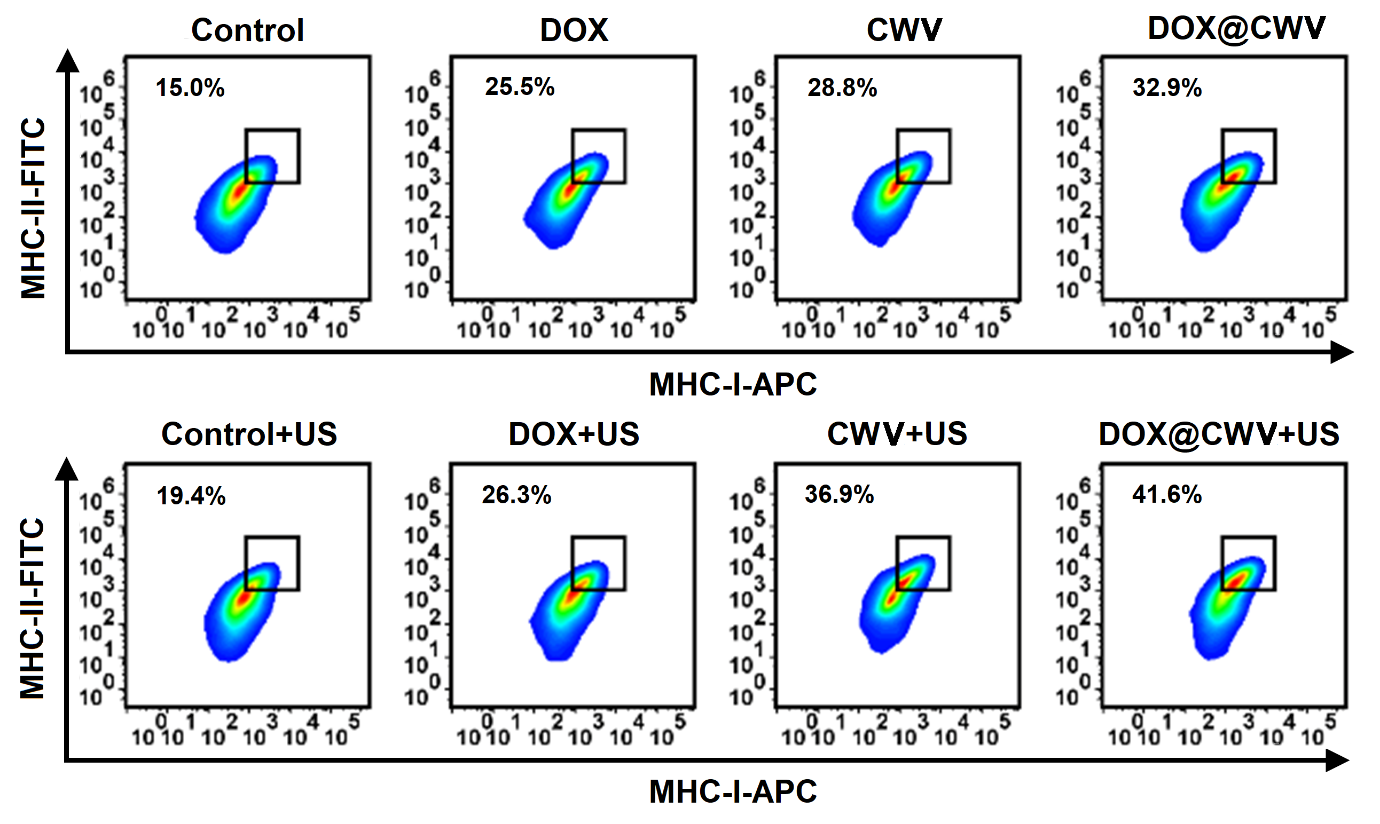


**Figure S26.** Flow cytometric plots of MHC-I^+^ MHC-II^+^ CD11c^+^ DCs in splenic tissues isolated from SCC-7 tumor-bearing mice after various treatments.


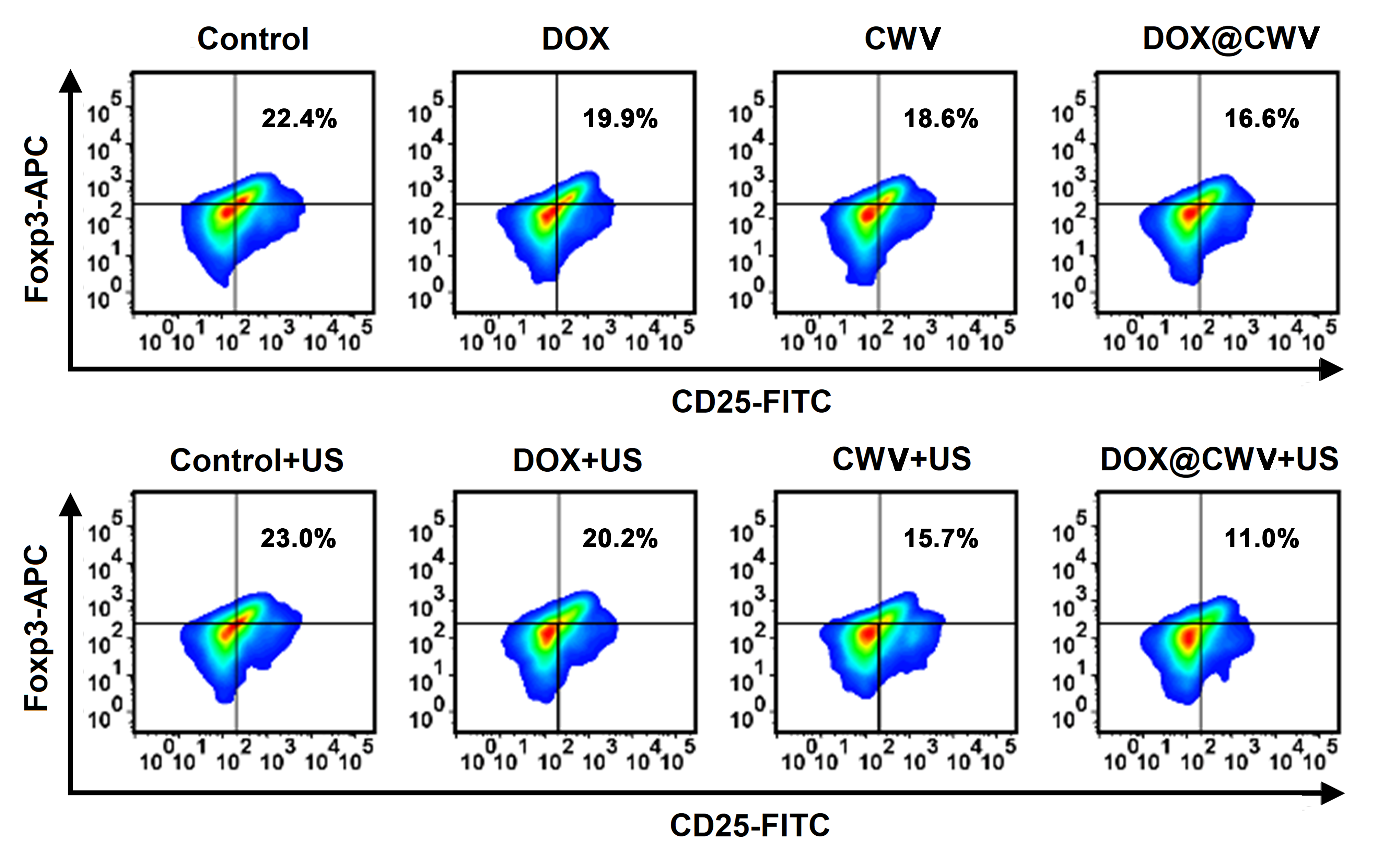


**Figure S27.** Flow cytometric plots of CD25^+^ Foxp3^+^ CD4^+^ T cells in splenic tissues isolated from SCC-7 tumor-bearing mice after various treatments.


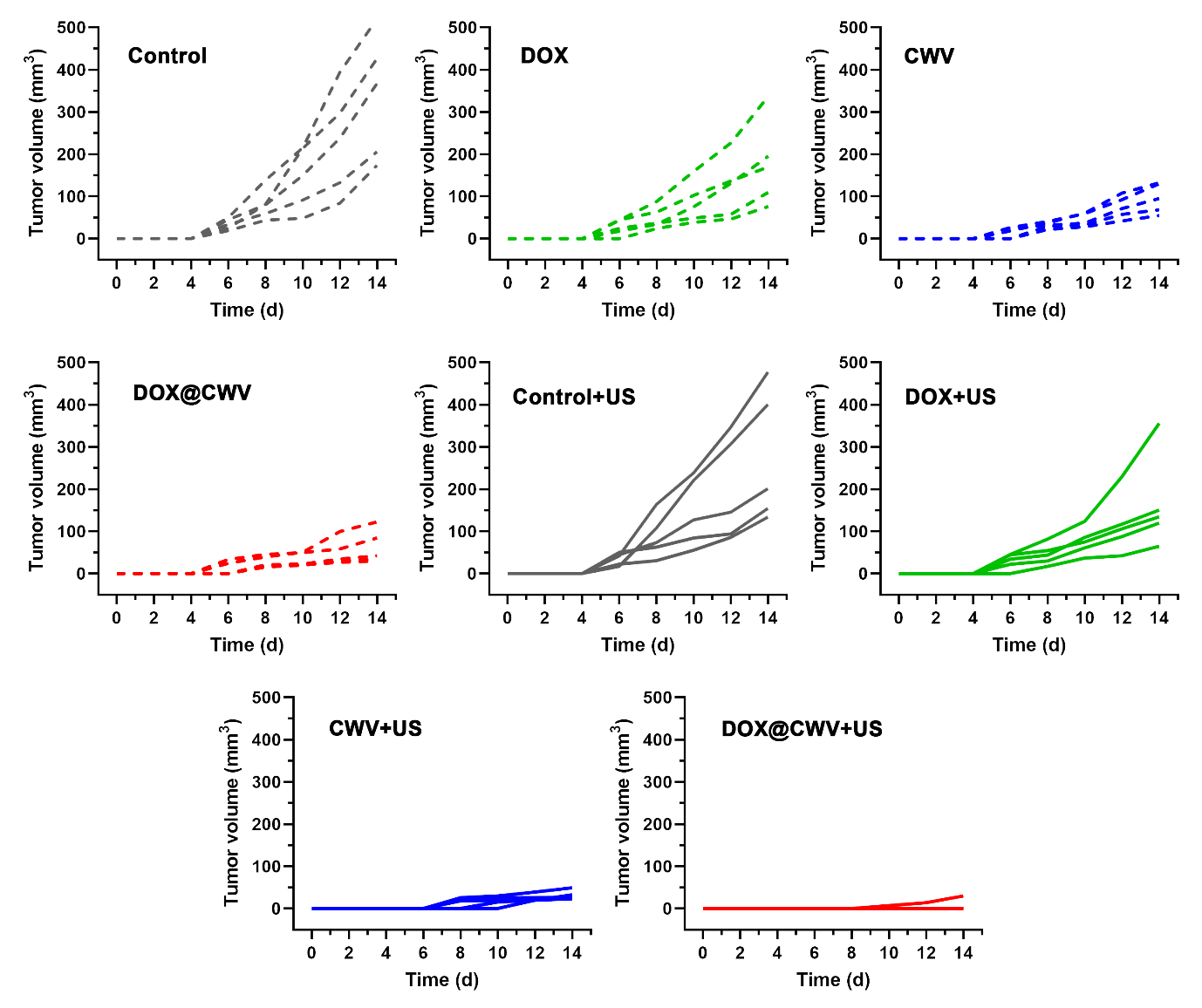


**Figure S28.** Individual growth curves of distant tumors from the mice bearing bilateral SCC-7 tumors within 14 d after the treatments of PBS ± US (the control ± US), DOX ± US, CWV ± US, and DOX@CWV ± US. The mice were administered with sample solutions via intratumoral injection. The doses of DOX and CWV were 0.15 mg kg^‒1^ and 7.5×10^7^ Pg cells kg^‒1^, respectively. US irradiation (1 MHz, 1.5 W cm^‒2^) was performed at the primary tumor site for 5 min.


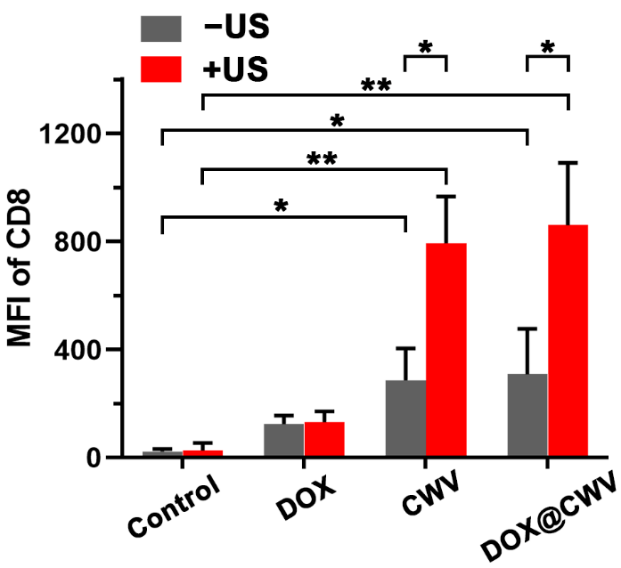


**Figure S29.** Comparison of the mean fluorescent intensity (MFI) of CD8 in distant tumor sections. Data are shown as mean values ± SD (n = 3). * and ** represent p < 0.05 and < 0.01 between two treatment groups (One-way ANOVA).


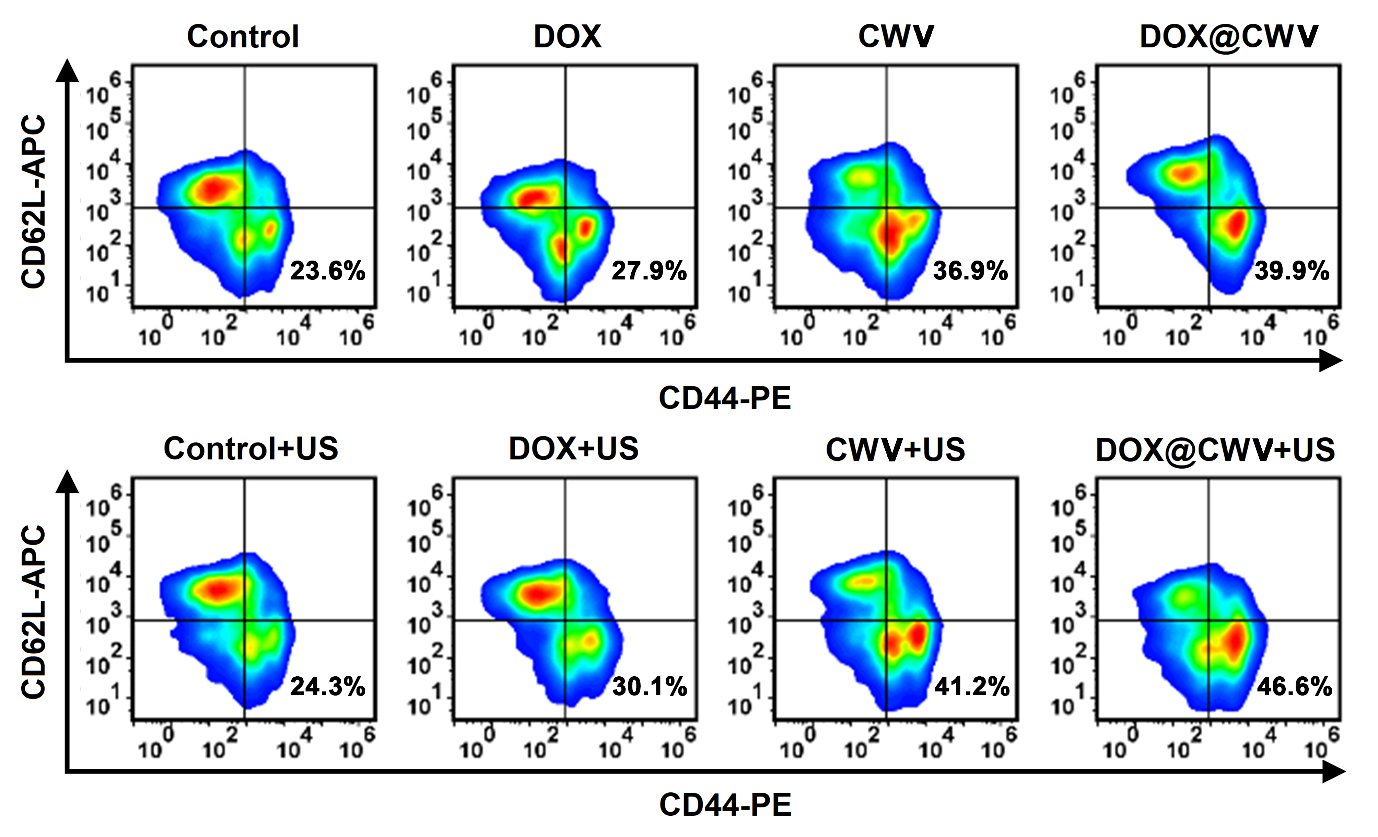


**Figure S30.** Flow cytometric plots of CD44^+^ CD62^–^ memory T cells in splenic tissues isolated of the mice bearing bilateral SCC-7 tumors after various treatments.
